# Partial and Full PCR-Based Reverse Genetics Strategy for Influenza Viruses

**DOI:** 10.1371/journal.pone.0046378

**Published:** 2012-09-28

**Authors:** Hongjun Chen, Jianqiang Ye, Kemin Xu, Matthew Angel, Hongxia Shao, Andrea Ferrero, Troy Sutton, Daniel R. Perez

**Affiliations:** Virginia-Maryland Regional College of Veterinary Medicine, Department of Veterinary Medicine, University of Maryland, College Park, Maryland, United States of America; University of Ottawa, Canada

## Abstract

Since 1999, plasmid-based reverse genetics (RG) systems have revolutionized the way influenza viruses are studied. However, it is not unusual to encounter cloning difficulties for one or more influenza genes while attempting to recover virus *de novo*. To overcome some of these shortcomings we sought to develop partial or full plasmid-free RG systems. The influenza gene of choice is assembled into a RG competent unit by virtue of overlapping PCR reactions containing a cDNA copy of the viral gene segment under the control of RNA polymerase I promoter (pol1) and termination (t1) signals – herein referred to as Flu PCR amplicons. Transfection of tissue culture cells with either HA or NA Flu PCR amplicons and 7 plasmids encoding the remaining influenza RG units, resulted in efficient virus rescue. Likewise, transfections including both HA and NA Flu PCR amplicons and 6 RG plasmids also resulted in efficient virus rescue. In addition, influenza viruses were recovered from a full set of Flu PCR amplicons without the use of plasmids.

## Introduction

Type A Influenza (Flu) viruses belong to the family Orthomyxoviridae and their genome consist of eight segments of single strand RNA of negative polarity [1]–[3]. The viral RNA (vRNA) is found in the virion and infected cells in the form of viral ribonucleoprotein particles (vRNPs) associated with three polymerase subunits (PB1, PB2, and PA) and the nucleoprotein (NP) [4], [5]. The virus encodes for two surface glycoproteins, hemagglutinin (HA) and neuraminidase (NA), the proton pump transmembrane protein (M2), the matrix protein (M1), the nuclear export protein (NS2/NEP), the nonstructural protein (NS1) and, in some influenza viruses (from an alternative translation start site in segment 1) the PB1-F2, an apoptosis modulatory protein [6]–[8]. Additional viral protein products include PB1-N40, derived from an alternative start site within the PB1 ORF, resulting in a protein product that lacks the first 39 aa of PB1, and PA-X, derived from the PA mRNA and consists of the N-terminal 191 aa of PA fused to 61 aa that result from +1 frameshifting [9], [10].

*De novo* synthesis of influenza viruses by reverse genetics (RG) requires not only the viral RNA but also the viral protein components [11]–[14]. Thus, RG systems for influenza rely invariably on a dual promoter concept: One for the synthesis of vRNA segments and another for the synthesis of viral mRNAs [11]. Since the termini of influenza vRNAs are crucial for virus replication, plasmids carrying a RNA polymerase I (pol1) or T7 RNA polymerase promoters have been used to generate vRNAs with the exact 3′ end, whereas a pol1 terminator sequence (t1) or a hepatitis ∂ ribozyme have been used to generate the exact 5′ end. Plasmids carrying typical RNA polymerase II (pol2) promoters (CMV and/or chicken β-actin promoters) have been utilized for the synthesis of influenza mRNAs [15]–[19]. Despite the great advantages of this technology, and although variations to the plasmid-based approach have been developed, they inevitably rely on a cloning step [20], [21]. A system that does not rely on cloning could speed up studies on the significance of mutations in the viral genome for replication and/or modulation of virulence. In this report, Flu PCR amplicons, instead of plasmids, are an efficient and viable alternative to the plasmid-based RG system.

## Results

### A Flu reporter PCR amplicon results in reporter activity

**Figure 1:**
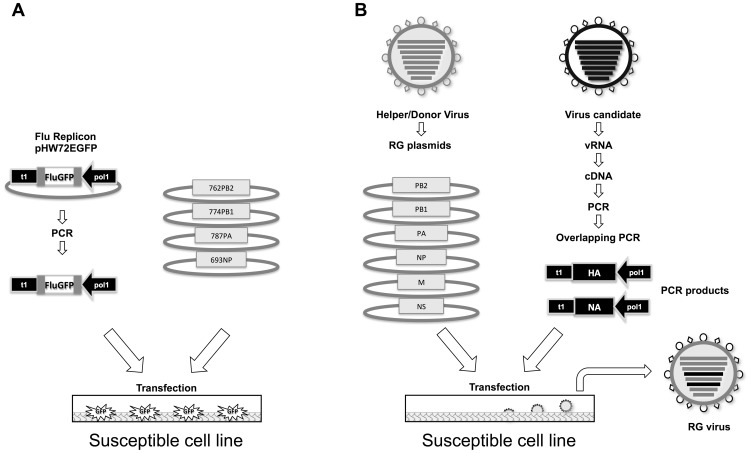
PCR-based reverse genetics. A) PCR-based Flu reporter replicon encoding GFP: PCR amplification was performed using primers spanning the pol1 to t1 sequences and pHW72EGFP. After agarose gel purification and testing to show that the PCR product is devoid of plasmid DNA contamination, the Flu GFP amplicon is transfected into 293T cells along with four expression plasmids encoding the polymerase complex of influenza virus. Expression of GFP reflects influenza polymerase activity on a vRNA Flu GFP replicon generated from pol1 transcription of the Flu GFP amplicon. Variations to this these are described in the main text and shown in Fig. 2. B) Starting with a influenza virus candidate, vRNA, cDNA and reconstitution of a full-length Flu PCR amplicon (in this case, the HA and NA PCR amplicons are depicted) is performed. Transfection of Flu PCR amplicons along with appropriate complementary RG plasmids into susceptible cells leads to the generation of recombinant influenza viruses with the desired gene constellation. The strategy speeds up the reverse genetics process by obviating a classical cloning step, which is currently part of the plasmid-based RG system.

**Figure 2:**
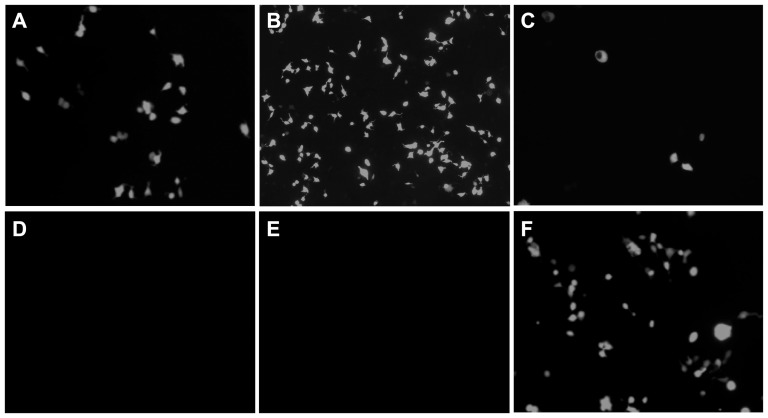
A Flu reporter PCR amplicon results in reporter activity. 293T cells were co-transfected with A) *polEGFPt1 PCR amplicon* and 4 plasmids expressing the influenza virus replication complex (3P - PB2, PB1, PA - and NP), B) pHW72-EGFP plasmid and 3P and NP plasmids, C) *pol1EGFPutr* and 3P and NP plasmids, D) *UTREGFPutr* (without *hpol1* and *t1* sequences) and 3P and NP plasmids, E) pHW72-EGFP plasmid and 2P (minus PB1) and NP plasmids, and F) *pol2PB2bgh*, *pol2PB1bgh*, *pol2PAbgh*, and *pol2NPbgh* amplicons and pHW72-EGFP plasmid. Pictures obtained at 24 hpt (20× magnification using the Carl Zeiss Axiophot Photomicroscope and UV filter). The amplicons were amplified by the method described in the supplemental information.

Flu GFP PCR amplicons were derived from pHW72-EGFP [21]. In order to determine whether a Flu PCR amplicon could be transfected into cells and be amplified by the influenza polymerase complex, a PCR product was produced encoding the GFP reporter gene in negative orientation flanked by the influenza segment 7 untranslated regions (UTRs) and further flanked by the human pol1 promoter and the mouse t1 termination signal, *pol1EGFPt1* (Fig. 1A, Fig. S1A, Table S1). Co-transfection of the *pol1EGFPt1* amplicon along with 4 protein expression plasmids encoding the influenza virus polymerase complex (3P) and NP into 293T cells resulted in efficient amplification of the reporter replicon and detection of green fluorescence signal (Fig. 2A). The proportion of green cells observed was comparable to those observed in the positive control cells co-transfected with pHW72-EGFP and the 3P and NP expression plasmids (Fig. 2B). The fluorescence signal of another amplicon, *pol1EGFPutr*, which lacks the t1 signal, was present in fewer cells compared to the *pol1EGFPt1* amplicon indicating that run off transcription by the RNA pol1 complex may result in vRNA fragments with incorrect and/or incomplete influenza sequences (Fig. 2C). As expected, no fluorescence signal appeared when cells were transfected with a Flu GFP PCR amplicon lacking the pol1 and t1 elements (*UTREGFPutr*) (Fig. 2D) or by removing the PB1 plasmid in co-transfected cells with either PCR amplicons or pHW72-EGFP plasmid (Fig. 2E and data not shown).

These initial studies were expanded in order to test whether PCR amplicons containing RNA polymerase II (pol2) and polyadenylation sequences flanking an appropriate ORF would result in gene expression (Fig. S1B). Thus, PCR amplicons of the 3P and NP genes were obtained using a set of primers spanning the cytomegalovirus promoter (CMV) and bovine growth hormone (bgh) polyA elements. Co-transfection of the *pol2PB2bgh*, *pol2PB1bgh*, *pol2PAbgh*, and *pol2NPbgh*, along with pHW72-EGFP, resulted in efficient reporter replicon expression indicating that PCR amplicons with either pol1 or pol2 transcription elements are appropriately transcribed by the corresponding transcription complexes (Fig. 2F).

### Generation of Flu PCR amplicons by overlapping PCR

**Figure 3:**
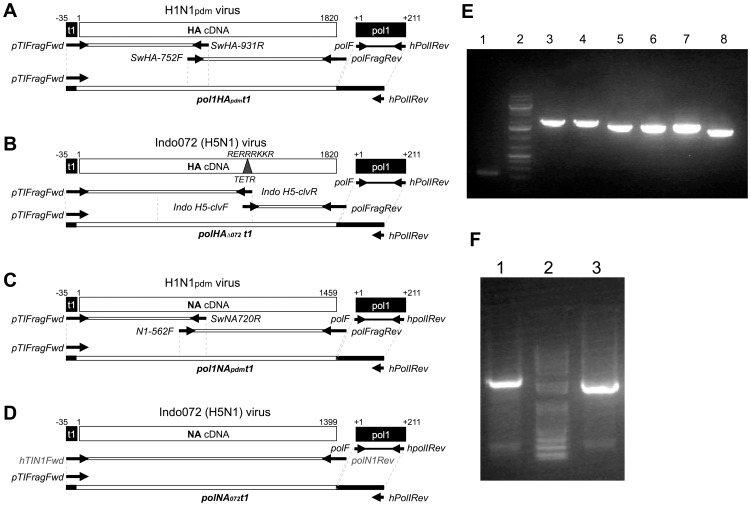
Overlapping PCR strategy and reconstitution of HA and NA PCR amplicons. The strategy to produce full-length HA and NA PCR amplicons was based on amplification of the pol1 promoter from the pDP2002 vector, subtype specific internal primers for HA and NA and, depending on the product, primers containing a t1 termination signal. A) The HA_pdm_ was amplified with the following overlapping fragments: 1, fragment spanning the primer set *pT1FragFwd* and *SwHA-931R* (which incorporates the *t1* signal), 2, fragment spanning from the *SwHA-752F* and *polFragRev*, and 3, *polI* promoter fragment amplified using *polF* and *hPol1Rev* primers. The three PCR products above were purified by agarose gel electrophoresis and combined in equal proportions (10 ng each) to create the full length *pol1HA_pdm_t1* PCR amplicon using the primer pair *pT1FragFwd* and *hPol1Rev*. B) An overlapping PCR strategy to produce an H5 *HA* segment in which the polybasic cleavage site from the chicken/North Sumatra strain was removed and replaced by the sequence of low pathogenic virus following the strategy described above. C) and D) Depict the strategies used for generation of full length N1 PCR products from the H1N1_pdm_ and 072 H5N1 strains. E) Alternative PCR products were generated to serve as controls for PCR-based reverse genetics. Agarose gel shows the expected size for lane 1, *hpol1* PCR amplicon; lane 2, GeneRuler™ 1 kb plus DNA Ladder; lane 3, *pol1HA_pdm_t1*; lane 4, *pol1HA_pdm_utr*; lane 5, *pol1NA_pdm_t1*; lane 6, *pol1NA_pdm_utr*; lane 7, *UTRHA_pdm_utr*; and lane 8, *UTRNA_pdm_utr*. F) *polHA*_Δ*072*_ *t1* (lane 1) and *polNA*_Δ*072*_ *t1* (lane 3) amplicons were prepared following the strategy described above and depicted in B) and D), respectively. Lane 2 corresponds to GeneRuler™ 1 kb Plus DNA Ladder (75–20,000 bp) from Fermentas Inc (lane 2). All the PCR products were amplified here and in the supplemental information.

In order to demonstrate whether Flu PCR amplicons could be used to replace plasmids in the RG system, the strains mouse-adapted A/California/04/2009 (H1N1) [22] and A/chicken/North Sumatra/072/2010 (H5N1) - herein referred to as H1N1pdm and 072, respectively - were used as donors for the HA and NA genes (Fig. 1B and Fig. 3). A specific set of internal primers designed within conserved regions of these gene segments were then developed in order to maximize gene amplification from viral cDNA preparations and to assemble the appropriate HA and NA PCR amplicons (Fig. 3, Fig. S 2A and B). The *pol1HA_pdm_t1* PCR amplicon carried a full-length copy of the HA gene from the H1N1pdm strain flanked by the *pol1* and *t1* signals (Fig. 2A). With respect to the 072 HA gene, the internal primers were designed to delete (Δ) the polybasic amino acid signal sequence (RERRKRRR) and replace it with one carrying a monobasic cleavage site (TETR) (Fig. 3B, Fig. S 2). Similar strategies were followed to create the NA amplicons *pol1NA_pdm_t1* and *polNA_072_t1* from viral cDNAs (Fig. 3C and D). Full-length *pol1HA_pdm_t1* and *pol1NA_pdm_t1* PCR amplicons were obtained and confirmed by sequencing (Fig. 2E). In addition, full-length HA and NA PCR amplicons were generated lacking either the t1 sequence or both the pol1 and t1 sequences, which serve as controls for efficiency of virus rescue as described below. Sequencing results confirmed the amplification of an overlapping ΔH5 HA amplicon, *pol1HA_Δ072_t1*, with a deleted polybasic cleavage site and the full-length amplification of the *polNA_072_t1* (Fig. 3F, Fig. S 2).

### Efficient influenza virus rescue using Flu PCR amplicons in either “1+7” or “2+6” modes

The *pol1HA_pdm_t1* or *pol1HA_Δ072_t1* HA PCR amplicons (Table 1) were co-transfected into co-cultured 293T/MDCK cells in a “1+7” mode along with 7 RG plasmids encoding the corresponding additional gene segments from the influenza A/Puerto Rico/8/1934 (H1N1) strain (PR8). At 48 h and 72 h post-transfection (hpt) cells co-transfected with the *pol1HA_pdm_t1* PCR amplicon plus 7 RG PR8 plasmids (H1_pdm_:7PR8) showed typical virus-induced cytopathic effect (CPE). H1_pdm_:7PR8 virus titers at 72 hpt reached 3.16×10^4^ TCID_50_/ml, which was 5 times lower than the one obtained using the corresponding pH1pdm RG plasmid (pH1_pdm_:7PR8, 1.58×10^5^ TCID_50_/ml) (Table 1). After a subsequent blind passage in MDCK cells or 9 day-old embryonated chicken eggs, virus titers increased >10^7^ TCID_50_/ml with either the *pol1HA_pdm_t1* PCR amplicon or the whole plasmid-based RG system. Likewise, the ΔH5N1 virus could be rescued using the *pol1HAΔ_072_t1* HA PCR amplicon and 7 RG PR8 plasmids (H5Δ_072_:7PR8, Table 1). At 72 hpt, H5Δ_072_:7PR8 virus titer in transfected cells was 1.58×10^4^ TCID_50_/ml, and increased to 2.32×10^8^ TCID_50_/ml when passaged in eggs (Table 1). At 72 hpt, the TICD_50_ titer of the PCR-based H5Δ_072_:7PR8 virus was 10, 000 times less than that of pH5Δ_072_:7PR8 virus, which was obtained with the bidirectional pRF1437 plasmid (Table 1).

**Table 1:** Virus rescue with Flu PCR amplicons in 293T/MDCK co-cultured cells.

Mode	Reassortant	PCR amplicons	Backbone/N° Plasmids	Transfectants (72 hpt)	Blind passage in MDCK (M) or embryonated eggs (E) (72 hpt)
	H1_pdm_:7PR8	*pol1HA_pdm_t1*	PR8/7	3.16×10^4^	1.58×10^7^ (M)
	pH1_pdm_:7PR8	-	pH1pdm/1, PR8/7	1.58×10^5^	2.32×10^7^ (M)
	H5Δ_072_:7PR8	*pol1HA_Δ072_t1*	PR8/7	1.58×10^4^	2.32×10^8^ (E)
	H1_pdm_ utr:7PR8^a^	*pol1HA_pdm_ utr*	PR8/7	2.00×10^2^	1.58×10^5^ (M)
	pH5Δ_072_:7PR8	*-*	PR8/7	1.58×10^8^	Not Done
7+1	UTRH1_pdm_utr:7PR8^b^	*UTRHA_pdm_utr*	PR8/7	0	0 (M); 0 (E)
	H1_pdm_:7WF10	*pol1HA_pdm_t1*	WF10/7	1.58×10^6^	Not Done
	pH1_pdm_:7WF10	*-*	pH1pdm/1, WF10/7	2.32×10^6^	Not Done
	H5Δ_072_:7WF10	*pol1HA_Δ072_t1*	WF10/7	1.58×10^6^	1.58×10^9^ (E)
	H1_pdm_7AA60_ca_	*pol1HA_pdm_t1*	AA60_ca_/7	0	0 (M); 0.50×10^3^ (E)
	pH1_pdm_:7AA60_ca_	*-*	pH1pdm/1, AA60_ca_/7	0	0 (M); 1.08×10^3^ (E)
	H5Δ_072_:7AA60_ca_	*pol1HAΔ_072_t1*	AA60_ca_/7	0.16×10^3^	1.08×10^5^ (E)
	H1N1_pdm_:6pdm	*pol1HA_pdm_t1/pol1NA_pdm_t1*	H1N1_pdm_/6	1.00×10^5^	2.32×10^7^ (M)
	H1N1_pdm_utr:6pdm	*pol1HA_pdm_utr/pol1NA_pdm_utr*	H1N1_pdm_/6	3.16×10^3^	1.85×10^6^ (M)
	UTRH1N1_pdm_utr:6pdm	*UTRHA_pdm_utr/UTRNA_pdm_utr*	H1N1_pdm_/6	0	0 (M); 0 (E)
	H1N1_pdm_:6PR8	*pol1HA_pdm_t1/pol1NA_pdm_t1*	PR8/6	3.16×10^4^	1.58×10^7^ (M)
	pH1N1_pdm_:6PR8	*-*	pH1N1_pdm_/2, PR8/6	1.58×10^5^	2.32×10^7^ (M)
	H5Δ_072_N1:6PR8	*pol1HA_Δ072_t1/pol1NA_072_t1*	PR8/6	1.58×10^4^	5.00×10^8^ (E)
6+2	H1N1_pdm_utr:6PR8	*pol1HA_pdm_utr/pol1NA_pdm_utr*	PR8/6	1.00×10^2^	2.34×10^5^ (M)
	UTRH1N1_pdm_utr:6PR8	*UTRHA_pdm_utr/UTRNA_pdm_utr*	PR8/6	0	0 (M); 0 (E)
	H1N1_pdm_:6WF10	*pol1HA_pdm_t1/pol1NA_pdm_t1*	WF10/6	1.08×10^5^	Not Done
	pH1N1_pdm_:6WF10	-	pH1N1_pdm_/2, WF10/6	1.58×10^6^	Not Done
	H5Δ_072_N1:6WF10	*pol1HA_Δ072_t1/pol1NA_072_t1*	WF10/6	1.08×10^5^	5.00×10^9^ (E)
	H1N1_pdm_:6AA60_ca_	*pol1HA_pdm_t1/pol1NA_pdm_t1*	AA60_ca_/6	0	0 (M); 2.32×10^4^ (E)
	pH1N1_pdm_:6AA60_ca_	-	pH1N1_pdm_/2, AA60_ca_/6	0	0 (M); 5.00×10^4^ (E)
	H5Δ_072_N1:6AA60_ca_	*pol1HA_Δ072_t1/pol1NA_072_t1*	AA60_ca_/6	<1	0.50×10^3^ (E)
4+4	4PCR:4PR8	*pol1HA_pdm_t1/pol1NA_pdm_t1/pol1NS_PR8_t1/polM_PR8_t1*	PR8/4	5.00×10^3^	1.08×10^7^ (M)
0+8	8PCR:3P/NP (PR8)	*pol1HA_pdm_t1/pol1NA_pdm_t1/pol1NS_PR8_t1/polM_PR8_t1/pol1PB2_PR8_t1/pol1PB1_PR8_t1/pol1PA_PR8_t1/pol1NP_PR8_t1*	PR8/4	1.58×10^2^	1.08×10^6^ (E)
0+12	12PCR (PR8)	*pol1HA_pdm_t1/pol1NA_pdm_t1/pol1NS_PR8_t1/polM_PR8_t1/pol1PB2_PR8_t1/pol1PB1_PR8_t1/pol1PA_PR8_t1/pol1NP_PR8_t1/pol2PB2bgh/pol2PB1bgh/pol2PAbgh/pol2NPbgh*	PR8/0	<1	1.58×10^6^ (E)

a utr: Viruses obtained from Flu PCR amplicons lacking a t1 termination signal.

b UTR: No viruses obtained when Flu PCR amplicons lacked the pol 1 promoter sequence.

**Figure 4:**
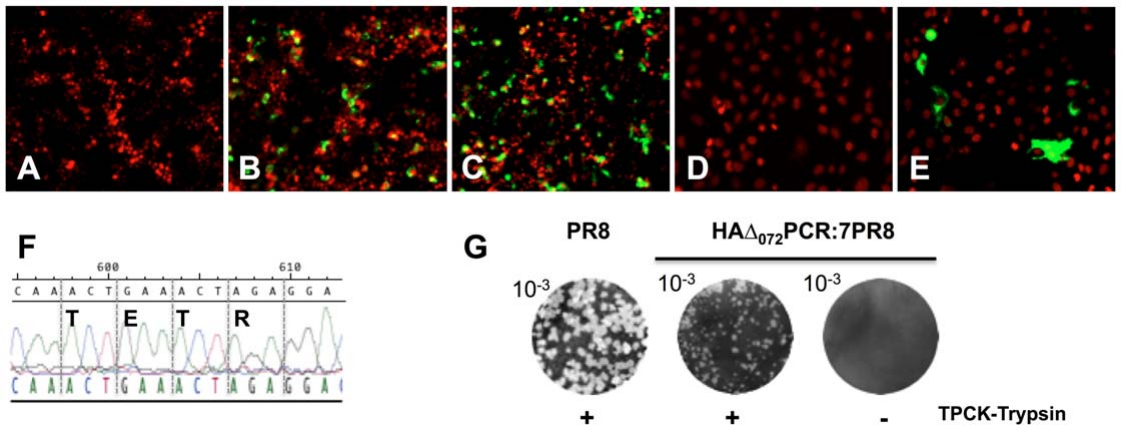
Identification of virus reassortants obtained using Flu PCR amplicons. Viruses recovered by reverse genetics were used to infect MDCK cells at a MOI of 0.001. At 36 hpi, cells were fixed with 4% Paraformaldehyde in PBS solution (Santa Cruz) and viral antigen detected by IFA using subtype specific monoclonal antibodies and FITC-labeled antimouse antibodies (green). Counterstaining was performed with propidium iodide (red). A) mAb 3B2 specific for the HA H1N1_pdm_ virus has no reaction with the HA of PR8 strain (only cell nuclei can be seen in red reacting with propidium iodide) (X10). B) and C) Positive viral antigen green signal detected using mAb 3B2 in cells infected with either the H1N1_pdm_utr:6PR8 or H1N1_pdm_:6PR8 viruses, respectively (X10). D) mAb DPJY01 specific to H5N1 virus has no reaction with PR8-infected cells (X20). E) Positive reaction of mAb DPJY01 cells infected with H5Δ_072_N1:6PR8 (X20). F) Sequence of HA cleavage site of H5Δ_072_N1:6PR8 virus indicates the presence of a low pathogenic pattern (TETR), which was created by overlapping PCR as indicated in the materials and methods and Fig. 3B. G) Plaque assay was performed as described in materials and methods. The PR8 and H5Δ_072_:7PR8 viruses form plaques in agarose plates in the presence of 1 µg/mL TPCK-Trypsin but not in its absence.

The identity of the 1+7 reassortants was further confirmed by sequencing the HA gene, and by immunofluorescence assay (IFA) (Fig. 4A) and plaque assay (Fig. 4B). As expected, no CPE and no virus was detected after transfection of cells with 7 RG PR8 plasmids (and in which the plasmid encoding the HA segment was omitted, not shown). By plaque assay, the H5Δ_072_:7PR8 virus was found to form plaques in agar plates in the presence, but not in the absence, of 1 µg/mL TPCK-(Fig. 4C), consistent with the reconstitution of a monobasic cleavage site in the HA gene during PCR amplification. To further confirm that these viruses carry a ΔH5 gene and/or do not present additional mutations introduced during PCR amplification, each of these viruses was plaque-purified and the HA gene was sequenced and analyzed for 48 plaque-purified viruses for each of three RG viruses, H5Δ_072_:7PR8, pH5Δ_072_:7PR8 , and control RG-derived PR8. Every single virus isolate from the H5Δ_072_:7PR8 virus group contained a monobasic cleavage site identical to the one designed in the PCR primers. Mutations were identified elsewhere but in only 4 out of the 48 sequences analyzed for the H5Δ_072_:7PR8 virus and in each case corresponded to single nucleotide changes (Table S2). The mutations caused amino acids changes in 3 of them; however, these changes did not alter the antigenic make up of these viruses by HI assay (Table S2). No mutations were identified in HA sequences derived from plaques obtained from the RG-derived PR8 or pH5_Δ072_:7PR8 viruses (data not shown). These results highlight the high fidelity of the PCR approach.

Because the PR8 virus is a fully laboratory adapted strain and can be recovered very efficiently by RG, studies were performed to test whether the *pol1HA_pdm_t1* or *pol1HA*Δ*_072_t1* PCR amplicons could be recovered in the background of other RG systems including the A/guinea fowl/Hong Kong/WF10/1999 (H9N2) [23] and the cold-adapted A/Ann Arbor/6/1960 (H2N2) [24] strains, herein referred to as WF10 and AA60_ca_, respectively (Table 1). In addition, virus rescue was performed in the context of the H1N1pdm background (Table 1). Regardless of the RG virus background used, virus rescued was possible. This was particularly the case using the WF10 background in which high virus titers (1.58×10^6^ TCID_50_/ml) in the initial co-transfected cells were obtained with either *pol1HA_pdm_t1* or *pol1HA*Δ*_072_t1* HA PCR amplicons (H1_pdm_PCR:7WF10 and H5Δ_072_:7WF10, respectively, Table 1). When passaged into eggs, virus titers increased significantly, about 1,000 fold for the H5Δ_072_:7WF10 virus. Virus titers below or just at the limit of detection were observed in cells co-transfected in the context of 7 RG plasmids from the AA60_ca_ strain carrying either the *pol1HA_pdm_t1* (H1_pdm_:7AA60_ca_) or *pol1HA*_Δ*072*_ *t1* (H5Δ_072_:AA60_ca_) HA PCR amplicons or the control plasmid pH1pdm (pH1_pdm_:7AA60_ca_). However, a >1,000 fold increase in virus titers were observed after blind passage in eggs of supernatants of the AA60_ca_-based co-transfected cells from these reassortant groups (Table 1).

In the 2+6 mode, efficient virus rescue was also obtained. No statistical differences were observed in rescue efficiency between the Flu HA PCR amplicon plus 7 PR8 RG plasmids (H1_pdm_:7PR8) compared to the HA and NA amplicons and 6 PR8 RG plasmids (H1N1_pdm_:6PR8). If the WF10 background was used, co-transfection of the HA and NA amplicons resulted in approximately 10 fold less virus (H1N1_pdm_:6WF10) in the supernatant of transfected cells compared to using the HA amplicon alone (HA_pdm_:7WF10). A similar trend was observed when the AA60_ca_ background was used (compare reassortants H1N1pdm:6AA60_ca_ with HA_pdm_:7AA60_ca_). ΔH5N1 viruses were rescued from the 072 strain using the amplicons *pol1HA*_Δ*072*_ *t1* and *pol1NA_072_t1* co-transfected with either the PR8, WF10, or AA60_ca_ backgrounds with efficiencies similar to those obtained using the HA and NA amplicons from the H1N1pdm strain. It must be noted that reassortant viruses carrying 7 gene segments from either WF10 or AA60_ca_ encode a N2 NA subtype, which may have affected the rescue efficiency of the H1_pdm_ or ΔH5 HA gene segments. Nevertheless, the results showed that either the 1+7 or 2+6 strategies using Flu PCR amplicons is a suitable method to speed up the recovery of influenza viruses by RG.

### Low efficiency of virus rescue using Flu PCR amplicons lacking the t1 signal

Since the PCR strategy with pol1 and t1 signals was initially used, we wanted to determine whether amplicons lacking the *t1* signal could be better substrates for the generation and subsequent amplification of vRNA segments. PCR amplicons were prepared and designated as *pol1HA_pdm_utr* or *pol1NA_pdm_utr* using the overlapping PCR method mentioned above, and in which the t1 signal was omitted. Similarly, PCR amplicons for HA and NA lacking both the pol1 and t1 signals were prepared and used as controls. Using either the PR8 or H1N1pdm virus backgrounds, virus rescue was possible with HA and NA PCR amplicons lacking the t1 signal, although the rescue efficiency was 100∼200 fold lower than using the same amplicon with the t1 signal. After passage in MDCK cells, virus titers of these reassortants (HA_pdm_utr:7PR8, HA_pdm_utr:7pdm, H1N1_pdm_utr:6PR8, and H1N1_pdm_utr:7pdm) were increased although they were 10∼30 fold lower than those obtained with the full-length t1 signal-containing amplicons (HA_pmd_:7PR8, HA_pdm_:7pdm, H1N1_pdm_:6PR8, and H1N1_pdm_:7pdm, Table 1). These results are consistent with the previous observation using the Flu GFP amplicon lacking the t1 signal, which suggests that the presence of the t1 signal helps generate optimal full-length Flu PCR amplicons.

### Plasmid-free reverse genetics using PCR amplicons

To expand the potential use of PCR amplicons as a suitable surrogate system to recover influenza viruses without the use of plasmids, each virus segment was amplified to generate a full set of Flu PCR amplicons encoding each one of the viral segments (Fig. S 3). The optimal cocktail of eight PCR amplicons, based on the HA and NA genes of the H1N1_pdm_ and 6 other amplicons from the PR8 strain, consisted of *polPB1_PR8_t1* (1 µg), *polPB2_PR8_t1* (1 µg), *polPA_PR8_t1* (1 µg), *polNP_PR8_t1* (1 µg), *polHA_pdm_t1* (0.5 µg), *polNA_pdm_t1* (0.5 µg), *polM_PR8_t1* (0.5 µg), and *polNS_PR8_t1* (0.3 µg), along with the 3P (1 µg each) and NP (1 µg) expression helper plasmids. The mixture was transfected into co-cultured 293T/MDCK cells with a ratio of 500/1 and at a density of 5×10^5^ cells. It resulted in low efficiency virus rescue with a titer of 1.58×10^2^ TCID_50_ after 72 hpt with no detectable HA titer (Table 1, 8PCR:3P/NP (PR8) virus). Blind passage in MDCK cells, resulted in virus titers in the order of 1×10^6^ TCID_50_/ml with an HA titer of 128. These studies were further expanded to include a full set of PCR products in which the 3P and NP expression plasmids were replaced by the corresponding *pol2PB2bgh*, *pol2PB1bgh*, *pol2PAbgh*, and *pol2NPbgh* amplicons in a reaction including 12 PCR amplicons and no plasmids (Table S1, 12PCR (PR8) virus). Here again virus rescue was possible albeit at reduced levels and only detected after blind passage in MDCK cells. Variations to this theme in which 4 PCR amplicons were used to replace the corresponding plasmids (Table 1, 4PCR:4PR8 virus), resulted also in efficient virus rescue indicating that the PCR-based strategy is not limited to just the viral surface genes and it could be easily applied to other gene segments that may be reluctant to cloning.

### Virus rescue by PCR amplicons in Vero and MDCK cells

Because Vero and MDCK cells have been approved for influenza vaccine production, we investigated whether Flu PCR amplicon rescue, either in 1+7 or 2+6 modes, was possible in these cells. Vero cells co-transfected with the Flu HA (alone or in combination with the NA) PCR amplicon from the H1N1pdm strains and 7 (or 6) PR8 RG plasmids resulted in virus rescue that was observed at 120 hpt (∼10^2^ TCID_50_/ml) with about 500 fold lower efficiency compared to the whole plasmid-based system (Table 2). Blind passage of supernatants of Vero cells at 72 hpt into MDCK cells resulted in virus titers similar to those obtained using the whole plasmid RG system (around 10^7^ TCID_50_/ml).

**Table 2:** Virus rescue with H1N1_pdm_ PCR amplicons in Vero cells.

Mode	Reassortants	PCR amplicons	Backbone/Plasmids (N)	Transfectants (72 hpt)	Blind passage on MDCK (72 hpt)
7+1	H1_pdm_:7PR8	*pol1HA_pdm_t1*	PR8/7	1.08×10^2^	1.58×10^7^
7+1	pH1_pdm_:7PR8	-	pH1pdm/1, PR8/7	5.00×10^4^	2.32×10^7^
6+2	H1N1_pdm_:6PR8	*pol1HA_pdm_t1/pol1NA_pdm_t1*	PR8/6	0.50×10^2^	1.58×10^7^
0+8	8PCR:3P/NP (PR8)	*pol1HA_pdm_t1/pol1NA_pdm_t1/pol1NS_PR8_t1/polM_PR8_t1/pol1PB2_PR8_t1/pol1PB1_PR8_t1/pol1PA_PR8_t1/pol1NP_PR8_t1*	PR8/4	<1	<1
0+12	12PCR (PR8)	*pol1HA_pdm_t1/pol1NA_pdm_t1/pol1NS_PR8_t1/polM_PR8_t1/pol1PB2_PR8_t1/pol1PB1_PR8_t1/pol1PA_PR8_t1/pol1NP_PR8_t1/pol2PB2bgh/pol2PB1bgh/pol2PAbgh/pol2NPbgh*	PR8/0	<1	<1

Using the 1+7 approach, virus rescue was also possible in MDCK cells with HA PCR amplicons from two H5N1 strains, 072 and A/Viet Nam/1203/2004 (VN1203) (Table 3). In this case, HA PCR amplicons were prepared carrying the canine pol1 promoter (k9pol1) and termination signals (k9t1). Both HA genes were amplified using overlapping PCRs that removed the gene's polybasic cleavage site sequences (Fig. S 4). Co-transfections of the HA PCR amplicons and 7 k9pol1-driven RG plasmids from the VN1203 strain in MDCK cells resulted in virus rescue with titers of ∼10^5^ TCID_50_/ml at 120 hpt and 10^8^ TCID_50_/ml after blind passage in MDCK cells (Table 3, reassortant viruses HAΔ_072_:7VN1203 and HAΔ_VN1203_:7VN1203). Like in the previous transfection studies, removing the *k9t1* signal from the PCR products resulted in impaired virus rescue (reassortant viruses HAΔ_072_utr:7VN1203 and HAΔ_VN1203_utr:7VN1203), and removing both the *k9pol1* and *k9t1* sequences resulted in no virus rescue.

**Table 3:** Flu PCR amplicons rescued with the VN1203 backbone in MDCK cells.

Reassortants	PCR amplicons	Transfectants (72 hpt)	Blind passage (72 hpt)
H5Δ_072_:7VN1203	*k9pol1HA*Δ*_072_t1*	2.32×10^5^	1.58×10^8^
H5Δ_072_utr:7VN1203	*k9pol1HA*Δ*_072_utr*	1.58×10^2^	5.00×10^7^
UTRH5Δ_072_utr:7VN1203	*UTRHA*Δ*_072_utr*	<1	<1
H5Δ_VN1203_:7VN1203	*k9pol1HA*Δ*_VN1203_t1*	5.00×10^5^	2.32×10^8^
H5Δ_VN1203_utr:7VN1203	*k9pol1HA*Δ*_VN1203_utr*	1.08×10^3^	1.08×10^8^
UTRH5Δ_VN1203_utr:7VN1203	*UTRHA*Δ*_VN1203_utr*	<1	<1

## Discussion

In this report, a significant modification was introduced to the plasmid-based reverse genetics system [25], [26] for influenza based on PCR amplicons. In order to optimize and maximize amplification of the genes of interest, a strategy involving overlapping PCR fragments for each segment was designed and used in conjunction with a high fidelity polymerase and corresponding buffer, Phusion high-fidelity PCR master mix with GC Buffer (New England Biolabs). This enzyme performed the best in our hands, compared to seven other commercially available DNA polymerases (Supplementary materials and methods S1). The synthesis of full length Flu PCR amplicons implies producing overlapping PCR fragments with distinct differences in GC- versus AT-rich regions. The human and canine pol1 promoters are approximately 75% GC-rich whereas the HA segment is approximately 60% AT-rich (data not shown). The approach succeeded in producing overlapping PCR amplicons for the HA and NA segments from different subtypes, including H1N1pdm, H5N1, or H9N2 (not shown) and from the 6 internal gene segments of the PR8 strain by designing overlapping primers and optimizing the PCR conditions (Fig. S 2). The amount obtained in each reaction for full length Flu PCR amplicons was in the order of 1∼5 µg (Fig. 2), which is sufficient for transfection and virus rescue and comparable to the amount of plasmid DNA used for transfection in the conventional plasmid-based RG system (Table 1). For the H5N1 vaccine candidates, the polybasic cleavage site (RERRRKKR) in highly pathogenic strains was easily removed and replaced by a low pathogenic sequence (TETR) by virtue of adequate set of primers and overlapping PCR (Table S1, Fig. 3 and Fig. S 2). Plaque assays revealed that the rescued H5Δ_072_PCR:7PR8 virus was strictly dependent on the presence of TPCK-trypsin, which strongly suggests that the virus preparation was devoid of any wild type virus contaminant (Fig. 4C. This is not surprising since each overlapping PCR fragment is produced and purified independently prior to assembly the reaction to generate the full length PCR product. In addition, a low mutation rate was observed with approximately 8% of the plaque-purified viruses having a single nucleotide mutation. It is not fully understood at this stage whether those mutations are naturally present in the wild type 072 virus. Notably, the HA gene of clone 43 (Table S2) contained a mutation at aa position 151, which was shown related to a switch of receptor specificity from α 2,3 to α 2, 6 linked sialic acids [27]. Since the 072 virus used in this report has undergone more than one passage in eggs, it is possible that the homogeneity of its population had contributed to the finding of the small mutation rate among the H5Δ_072_PCR:7PR8 clones. Nevertheless, the results clearly show that the PCR-based RG strategy does not create a heterogeneous virus population. In this regard, it could be argued that the PCR-based approach will be most likely better represent the virus population present in the original virus isolated compared to the plasmid-based system.

Since it was not clear whether the presence of the t1 signal would have either a positive or negative effect on the production of full-length Flu PCR amplicons, it was important to determine whether run off pol1 transcription would make a difference in virus rescue efficiency using PCR amplicons. The results show that the presence of the t1 signal at the 3′ end of the Flu PCR amplicon greatly improved virus rescue efficiency, approximately 100–200 fold (Tables 1 and 3). This observation was consistent throughout the studies since omitting the t1 signal resulted in less efficient amplification of the Flu GFP replicon (Fig. 2C) and significantly less virus rescue in both human and canine cells (Tables 1 and 3).

The HA and NA PCR amplicons contributed to produce virus efficiently in the background of not only the laboratory adapted strain PR8 but also in other strains like the AA60_ca_ live attenuated vaccine strain or the WF10 and H1N1pdm wild type strains (Table 1). Furthermore, the approach is not limited to production of virus from transfected 293T or co-cultured 293T/MDCK cells. Transfection of Vero cells in a 1+7 and 2+6 mode also resulted in virus rescue (Table 2). Virus rescue of the ΔH5N1 1+7 virus in MDCK cells, with the attenuated HA PCR amplicons carrying the k9pol1 and k9t1 signals, was also successful (Table 3).

The boundaries of the system were extended by showing that a complete set of 8 Flu PCR amplicons can be effectively recovered by RG in the context of 4 expression plasmids encoding the influenza polymerase complex (Table 1). More importantly, an expression competent PCR version of the 3P and NP was also effective in rescuing the virus in a transfection reaction that contained no plasmids (Table 1). Our studies imply that protein expression studies could be performed without preparation of clones, although, it must be emphasize that systematic studies to evaluate the differences in protein expression of pol2-based PCR amplicons and their corresponding plasmids were not performed.

Although it can be argued that rescue efficiency was lower than using a plasmid-based approach, further optimization of the PCR-based system is likely possible by manipulating the amount and proportion of each amplicon in the transfection. Such analysis is beyond the scope of the present report. It could also be argued that the PCR-based system may produce a more variable virus population than the one that is obtained using the plasmid-based system. Although there is such possibility, it is an inherent nature of influenza viruses to evolve through point mutations and therefore no reverse genetics system is error free. However, the sequencing of reassortants produced in this study does not show mutations that would alter the antigenicity of the HA surface proteins. In this regard, for vaccine development, as long as the vaccine seed stock is antigenically identical to the vaccine candidate, other mutations would be irrelevant. In fact, inactivated influenza vaccines prepared by classical reassortment have only two pre-requisites: 1) HA surface gene derived from the vaccine candidate and 2) high growth in eggs. Full genome sequencing of vaccine viruses is not a pre-requisite for approval of the vaccine by the FDA.

Overall, the implications of the PCR-based approach for RG development are highly significant. Using a combination of PCR amplicons and plasmids, it would be possible to streamline the study of gene variants for one or more gene segments and determine fitness, pathogenesis or any other biological aspect of the virus. Several mutant viruses with mutations in one or more genes could be produced without having to prepare individual clones. Recovery of viruses entirely from PCR products implies that other viral systems could be amenable to a similar strategy. This could be particularly important for viruses with genomes larger than the influenza virus that are occasionally associated with cloning difficulties or plasmid instability. In fact, PCR amplification for the purpose of virus generation has already been done for coronaviruses, although the PCR product was used as template for in vitro RNA transcription and the resulting RNA was later transfected into cells [28]. In summary, a RG system for influenza was developed that does not require a cloning step for recovery of viruses and has profound implications for vaccine development, pandemic preparedness, and for the study of influenza viruses.

## Materials and Methods

### Viruses and cells

The mouse-adapted H1N1pdm virus and WF10 viruses have been previously described [22], [23]. The highly pathogenic H5N1 virus strain A/Chicken/NorthSumatra/Mdn/072/ 2010 (strain 072) was a gift from Teguh Prajitno (Vaksindo, Indonesia) and sent to us by Ron Fouchier (Erasmus Medical Center, the Netherlands). The VN1203 virus (H5N1 clade 1) was obtained from the Centers for Disease Control and Prevention, Atlanta, GA (CDC). The influenza PR8 strain was grown from a reverse genetics clone obtained from Peter Palese, Mount Sinai School of Medicine, New York, NY (H1N1) (PR8). Virus stocks were prepared in specific pathogen free 9-day old embryonated chickens eggs following standard techniques for growth of influenza viruses. MDCK and Vero cells were maintained in Modified Eagle's medium (MEM) (Sigma-Aldrich, St. Louis, MO) containing 5% fetal bovine serum (FBS) (Sigma-Aldrich). Human embryonic kidney cell-line 293T (HEK293T) were cultured in Opti-MEM I (GIBCO, Grand Island, NY) containing 5% FBS.

### Plasmids

The RG 8-plasmid system for the PR8 virus was a gift from Dr. Peter Palese, Mount Sinai School of Medicine, New York, NY. The RG system for the Ann Arbor cold adapted (ca) virus was produced from the homologous strain provided to us by Ruben Donis, CDC, and cloned into the pDP2002 vector (Sutton et al, unpublished). The RG 8-plasmid systems for the H1N1pdm and WF10 have been previously described [22], [29]. The bidirectional pGD2007 RG vector containing the canine pol1 and t1 sequences was a gift from Ruben Donis. The 8 gene segments of VN1203 were subcloned into the pGD2007 vector from clones originally prepared in the pDP2002 vector (data not shown). The plasmids pRF1428 and pRF1437 were provided by Ron Fouchier and encode the wt H5 HA and ΔH5 HA genes, respectively, from the 072 virus. The plasmids pcDNA774PB1, pcDNA762PB2, pcDNA787PA and pcDNA693NP have been previously described [30]. The pHW72-EGFP plasmid encoding the influenza EGFP reporter replicon was a gift from Robert Webster, St Jude Children's Research Hospital. Memphis, TN [21].

### Preparation of viral RNA and cDNA

The vRNAs and cDNAs from wild type and blind passage reassortant viruses were prepared as previously described [22], [31]. Briefly, total RNAs were extracted by using the RNeasy kit (Qiagen, Valencia, CA) following manufacturer's instructions. Reverse transcription was carried out with the Uni12 primer (5′-AGCAAAAGCAAGG-3′) and avian myeloblastosis virus (AMV) reverse transcriptase (Promega, Madison, WI). The cDNAs were stored at −80°C until use.

### PCR strategy for Flu EGFP replicon and pol2-driven P and NP expression PCR amplicons

The Flu EGFP replicon was amplified with the primers pT1FragFwd and hpol1Rev using pHW-EGFP plasmid DNA as the template [30]. PCR amplicons from pcDNA762 (PB2), pcDNA774 (PB1), pcDNA787 (PA) or pcDNA693 (NP) were generated with the primers pCMVF and pBGHR, and were designated as *pol2PB2bgh, pol2PB1bgh*, *pol2PAbgh* and *pol2NPbgh*, respectively [21], [30], [32]. The pol2-based Flu PCR amplicons were flanked by sequences corresponding to the immediate–early human cytomegalovirus (CMV) promoter and bovine growth hormone (*bgh*) polyadenylation signal. PCR conditions were similar to the overlapping PCR of *pol1HA_pdm_t1* and *pol1NA_pdm_t1* amplicons except for the use of 10 pg of the corresponding plasmid DNA template (see below).

After PCR amplification, two methods were used to demonstrate that PCR products were devoid of spurious plasmid DNA contamination. PCR products were digested with *Dpn* I (New England Biolabs, Ipswich, MA) for 1 h, and then separated and purified by agarose gel electrophoresis, and subsequently 100 ng of the purified PCR product were used to transform *E. coli* TOP10 cells (Invitrogen, Carlsbad, CA). Alternatively, the primer pairs, *pCMVF* and *pT1FragRev*, *pDP2066-2090F* and *pDP2392-2416R*, and *pT1-2F* and *UTR-H1Rev* were used to demonstrate lack of plasmid DNA contamination in purified PCR reactions (Table 1). This strategy was also used to demonstrate no plasmid DNA contamination after PCR amplification of the pol1 promoter from the RG vector pDP2002. In all instances, no plasmid DNA contamination was observed by either method (data not shown).

### PCR strategy of overlapping HA and NA gene segments with human pol1 promoter

A schematic representation of the overlapping PCR approach is shown in Fig. 2 using the set of primers described in Table S1. Two overlapping PCR fragments were generated for the HA_pdm_ gene: The first fragment spans from the primer set *pT1FragFwd*, which incorporates the t1 signal, and *SwHA-931R*. The second fragment was amplified with the primer pair *SwHA-752F* and *polFragRev*. The human pol1 promoter was PCR amplified using primers *polF* and *hPol1Rev* and the pDP2002 plasmid vector as template. Details of amplification conditions for each PCR fragment are provided with the supplementary information (Fig. S. 2A and B). The 3 PCR products above were purified by agarose gel electrophoresis and combined in equal proportions to generate a full-length HA PCR amplicon using the primer pair *pT1FragFwd* and *hPol1Rev*. The 50 µl PCR reaction mixture contained 10 ng of each PCR product, 25 µl of Master PCR mix, 1.5 µl 100% DMSO, and 50 pmol/µl of each primer. PCR reaction conditions were 98°C for 30 sec, and then 30 cycles at 98°C for 8 s, 56°C for 1 min and 72°C for 3 min, ending with 72°C for 10 min. PCR products were amplified using the Phusion high-fidelity PCR master mix with GC Buffer (New England Biolabs, Ipswich, MA). Alternative HA PCR products without t1 signal sequence or lacking both pol1 and t1 elements were generated to serve as controls for PCR-based reverse genetics (Fig. 2 and Table 2). PCR products were purified by agarose gel electrophoresis and quantitated after gel purification using Nanodrop 1000 (Nanodrop, Wilmington, DE). Overlapping PCR products were produced for the NA_pdm_ gene using the primer pair *pTIFragFwd* and *SwNA-763R* and *N1-562F* and *polFragRev*, whereas the full length NA PCR amplicon was generated with the primer pair *pT1FragFwd* and *hPol1Rev*. Alternative full-length NA amplicons were generated using specific primers as noted in Table 1.

Similar strategies were used to amplify the HAΔ_072_ and HAΔ_VN1203_ PCR products in which the polybasic cleavage site sequence were removed using overlapping PCR products spanning sequences from the primer pairs *pTIFragFwd and IndoH5-clvR and IndoH5-clvF* and *polFragRev* whereas the full length HA PCR amplicon as generated with the primer set *pT1FragFwd* and *hPol1Rev*. The full length NA gene segment from Indo072 strain was amplified without the generation of internal overlapping fragments using the primer set *hTIN1Fwd* and *polN1Rev* rather and then subsequently introduced in a PCR reaction to generate the full-length *pol1NA_072_t1* PCR amplicon carrying the pol1 promoter (Table S1). To compare the rescue efficiency of PCR amplicons, pRF1437 plasmid was also used as control.

### Overlapping PCR for the internal gene segments of PR8

To set up a reverse genetics system using a full set of PCR amplicons, the H1N1_pdm_ surface gene segments and the PR8 virus 6 internal gene segments were selected. The internal genes were amplified from the total cDNAs prepared from a wildtype PR8 strain, with an initial titer of 1.58×10^9^ TCID_50_/ml in MDCK cells. For the overlapping PR8 PB2 PCR amplicon with pol1 and t1 signal sequences, the pTIFragFwd and PB2-1811R primers were used to produce the N terminal fragment of PB2 (PB2-N), and then the PB2-1643F and polFragRev primers were used to produce the C terminal fragment (PB2-C). PB2-N, PB2-C, and pol1 fragments were mixed (10 ng each), and the overlapping PCR reaction was performed using primers pT1FragFwd and hPol1Rev. The PCR parameters were similar to those of *pol1HA_pdm_t1* and *pol1NA_pdm_t1* amplicons described above. The final product was labeled *pol1PB2_PR8_t1*. A similar strategy was followed for other gene segments fused to pol1 and t1 signal sequences, using the primer pairs: PB1-1240F/PB1-1531R, PA-892F/PA-1314R, HA-760F/HA-1274R, NP-1116F/NP-1441R, NA-743F/NA-905R, M-741F/M-915R, or NS-469F/NS-887R primers respectively. Final PCR amplicons were designated as *pol1PB1_PR8_t1*, *pol1PA_PR8_t1*, *pol1HA_PR8_t1*, *pol1NP_PR8_t1*, *pol1NA_PR8_t1*, *po1M_PR8_t1*, and *pol1NS_PR8_t1*, respectively. Additional details are provided with the supplementary information (Fig. S. 3).

### Overlapping PCR with canine *pol1* promoter

The generation of the HA PCR amplicon from H5N1 containing the k9pol1 promoter was performed as follows: The k9pol1 promoter was amplified from the pGD2007 vector using the primer pair *k9pol1F* and *k9pol1R* under these conditions: the initial denaturation at 98°C for 30 sec, 30 cycles of 98°C for 8 sec, 60°C for 30 sec, and 72°C for 1 min and the final extension at 72°C for 10 min (Table S1). Overlapping HA fragments, one containing the k9t1 signal was produced with the primer pair *kTIUni12F* and *IndoH5-clvR* (H5N1) and the second one with the primer pair *IndoH5-clvF* (H5N1) and *kPolUTRR* under the following conditions: pre-PCR treatment at 98°C for 30 sec, 30 cycles of 98°C for 8 sec, 56°C for 30 sec, and 72°C for 1 min and the final extension at 72°C for 10 min (Table S1). The amount of overlapping HA PCR products and the k9pol1 amplicons were calculated and mixed together at a concentration of 10 ng each. A full length HA PCR amplicon was generated with the primer pair *k9TIUni12F* and *k9Pol1R*. The thermal profile was: denaturation at 98°C for 30 sec, 30 cycles of 98°C for 8 sec, 56°C for 1 min, and 72°C for 4 min, and then extension at 72°C for 10 min. All the PCR products were amplified using the Phusion high-fidelity PCR master mix with GC Buffer.

### Generation of virus by reverse genetics using PCR amplicons

For partial plasmid-free rescue, the plasmid of choice was replaced with the corresponding Flu PCR amplicon and virus rescue performed essentially as described [26] with minor modifications. Briefly, co-cultured 293T/MDCK cells at a ratio of 500∶1 (5×10^5^ cells per well) was seeded into each well of a 6-well tissue culture plate. The plates were incubated at 37°C overnight. The following day, 1 µg of each plasmid or Flu PCR amplicons was incubated for 45 min with 16 µl of Transit-L1 transfection reagent (Mirus Bio LLC, Madison, WI ) and then the transfection allowed to occur overnight before the media was replaced with fresh serum-free Opti-MEM. At 24 h post-transfection (hpt), L-(tosylamido-2-phenyl) ethyl chloromethyl ketone (TPCK)-treated trypsin (1 µg/ml) was added to the cell supernatants.

MDCK and Vero cells were grown to 70% confluency in 75-cm^2^ flasks and then trypsinized with trypsin-EDTA (Invitrogen) and resuspended in Opti-MEM I containing 5% FBS. Cell suspensions were seeded into 6-well tissue culture plates and incubated at 37°C overnight before transfection. Transfections and post-transfection steps proceeded as described for the 293-T/MDCK co-cultured cells, except that Vero cells were incubated with 2 µg/ml of TPCK-trypsin.

Supernatant of transfected cells were collected at the times indicated in Tables 1, 2 and 3 and blind passage in either or both MDCK cells or 10-day old embryonated chicken eggs to monitor for the presence of rescued viruses. TCID_50_ titers were determined in MDCK cells by the Reed and Muench method as described [33]. Virus stocks were prepared and frozen at −80°C until use.

### Plaque staining and purification

The rescued viruses were examined by plaque assay in MDCK cells (30). Briefly, confluent cell monolayers in 6-well plates were infected with 10-fold dilutions of virus in a total volume of 0.4 ml PBS for 1 h at 37°C. Cells were washed twice with PBS and covered with an overlay of modified Eagle's medium containing 0.9% agar, 0.02% BSA, 1% glutamine, and, when noted, 1 ug/ml TPCK treated-trypsin. The plates were then incubated at 37°C under 5% CO_2_. After 3 days of incubation the overlays were removed and the cells were stained with 0.1% crystal violet.

For sequencing the HA gene from plaque-purified viruses, the agarose was plucked from areas of the plate were plaques were observed. Viruses were eluted from the agarose pluck in tissue culture media and later expanded in MDCK cells for 72 h prior to RNA extraction, cDNA synthesis, PCR, and sequencing.

### HI assay

Antisera against the ΔH5N1 Indo072 virus were raised in chickens by infecting them with a live attenuated version of the ΔH5N1 Indo072 virus in the background of the WF10att virus similar to as previously described [34]. Collected sera were treated with receptor-destroying enzyme (Accurate Chemical and Scientific Corp., Westbury, NY) prior to HI assays as described in the WHO Animal Influenza Training Manual (WHO/CDS/CSR/NCS/ 2002.5) (30).

### Sequence analysis

Sequencing of overlapping PCR products, viral cDNAs and HA sequences from the purified clones of PR8 or rescued reassortants was performed using a combination of universal primers [31] and custom made primers (available upon request) and the Big Dye Terminator v3.1 Cycle Sequencing kit (Applied Biosystems, Foster City, CA) on a 3500 Genetic Analyzer (Applied Biosystems, Foster City, CA) according to the manufacturer's instructions. Sequence analysis was performed using software available through the Lasergene package (DNAstar Inc., Madison, WI).

### Immunofluorescence assay

Cells grown in 96-well plates were infected with rescued influenza viruses at a dose of 1 TCID_50_/well. At 36 hpi, the cells were washed in precooled 0.01 M Phosphate Buffered Saline (PBS) buffer and fixed in neutral formaldehyde for 20 min at room temperature. The cells were then incubated with blocking solution (10% normal goat serum in PBS) for 1 h and probed with a primary antibody for 30 min. Two monoclonal antibodies were used to identify the recombinant influenza viruses: mAb 3B2 is specific for the HA protein of H1N1pdm viruses and which does not react with the HA of PR8 and other subtypes of influenza A viruses [35]. mAb DPJY01 is specific for the HA of H5 subtype influenza viruses [36]. The antibody-antigen complexes were further incubated with fluorescein isothiocyanate (FITC)-conjugated goat anti-mouse Ig (H+L) (Southernwest Biotech Associates Inc, Birmingham, AL) for 30 min at room temperature. The cells were washed three times with PBS after incubation and then counterstained with propidium iodide (PI) and examined under an Axiophot Photomicroscope produced by Carl Zeiss (λEx of 488/543 nm, λEm of 522/590 nm for 100 ms).

## Supporting Information

Materials and Methods S1 **Enzymes and kits used to generate full-length Flu PCR amplicons.**
(DOC)

Figure S1 **Pol1- and pol2-driven PCR amplicons.** A) Generation of Flu EGFP replicons from pHW72EGFP. Lane 1, pol1EGFPt1 amplicon amplified with the primer pair pT1FragFwd hpol1Rev. The Flu EGFP amplicon (1103 bp) contained the Flu EGFP replicon (846 bp) flanked by the human pol1 (222 bp) and mouse t1 (35 bp) sequences. Lane 2, pol1EGFPutr amplicon (1068 bp, lacking the t1 sequence) produced with the primer pair Bm-M-1F and hpol1Rev. Lane 3, UTREGFPutr amplicon (846 bp, lacking the pol1 and t1 sequences) amplified with the primers Bm-M-1F and Bm-M-1043R. Lane 4, UTREGFPt1 amplicon (881 bp, lacking pol1 sequence) generated with the primers pT1FragFwd and pol1FragRev. B) Pol2 Flu PCR amplicons produced from pcDNA762 (PB2), pcDNA774 (PB1), pcDNA787 (PA) and pcDNA693 (NP), respectively using the primer pair *pCMVF* and *pBGHR*. Each pol2 Flu PCR amplicon contained the cytomegalovirus immediate early promoter sequence (CMV, 659 bp), the bovine growth hormone polyA signal (BGHpA, 228 bp) and additional non coding regions present within the multiple cloning site of pcDNA3 (Invitrogen). Lane 1, *pol2PB2bgh* (3,386 bp); lane 2, *pol2PB1bgh* (3,385 bp); lane 3, *pol2PAbgh* (3,271 bp); and lane 4 *pol2NPbgh* (2,603 bp). “M” in panels A and B corresponds to DNA molecular weight marker (GeneRuler™ 1 kb Plus DNA Ladder, Fermentas).
(JPG)

Figure S2 **Generation of HA and NA amplicons.** cDNAs from the H1N1pdm and H5N1 072 viruses were prepared as described in the main text. M, GeneRuler™ 1 kb plus DNA Ladder. Lane 1, unspecific PCR products obtained using one-step RT-PCR to generate the full length of *HA_pdm_* gene (1,840 bp) with the primers pT1HF and polHR. Lane 2, the N terminus of *HA_pdm_* specific PCR product (998 bp) obtained using the primer pair pT1FragFwd, which incorporates the t1 signal, and SwHA-931R. Lane 3, the C terminus of overlapping *HA_pdm_* specific PCR product (1,022 bp) using the primer pair SwHA-752F and polFragRev. Lane 4, the N terminus of *NA_pdm_* specific PCR product (799 bp) obtained using the primer pair pTIFragFwd and SwNA-763R. Lane 5, the C terminus of *NA_pdm_* specific PCR product (924 bp) from N1-562F and polFragRev primer set. Lane 6, the first *HA*Δ*_072_* specific PCR fragment (1,090 bp) obtained with the primers pTIFragFwd and IndoH5-clvR. Lane 7, the second *HA*_Δ*072*_ specific PCR fragment (762 bp) obtained with the primers pair IndoH5-clvF and pol1FragRev. Lane 8, the full-length *NA*_Δ*072*_ amplicon (1,460 bp) generated with the primer set hTIN1Fwd and polN1Rev. The 25 µl PCR reaction mixture contained 10 ng of cDNAs, 12.5 µl of Master PCR mix, 0.6 µl 100% DMSO, and 10 pmol/µl of each primer. The PCR reaction conditions were 98°C for 30 sec, and then 30 cycles at 98°C for 8 s, 56°C for 1 sec and 72°C for 2 min, ending with 72°C for 10 min. PCR products were amplified using the Phusion high-fidelity PCR master mix with GC Buffer.
(JPG)

Figure S3 **Full-length PCR amplicons from PR8 virus gene segments.** A) The PR8 virus gene segments were amplified as two overlapping PCR fragments, which were performed as follows: Lane 1, amplification of the N terminal fragment of *PB2_PR8_* (1,846 bp) with primer pair pTIFragFwd and PB2-1811R. Lane 2, amplification of the C terminal fragment of *PB2_PR8_* (741 bp, yellow arrow) with primer pair PB2-1643F and polFragRev. Lane 3, amplification of the N terminal fragment of *PB1_PR8_* (1,566 bp) with primer pair pTIFragFwd and PB1-1531R. Lane 4, amplification of the C terminal fragment of *PB1_PR8_* (1,128 bp) with primer pair PB1-1240F and polFragRev. Lane 5, amplification of the N terminal fragment of *PA_PR8_* (1,349 bp) with primer pair pTIFragFwd and PA-1314R. Lane 6, amplification of the C terminal fragment of *PA_PR8_* (1,368 bp) with primer pair PA-892F and polFragRev. Lane 7, amplification of the N terminal fragment of *HA_PR8_* (1,309 bp) with primer pair pTIFragFwd and HA1274R. Lane 8, amplification of the C terminal fragment of *HA_PR8_* (1,042 bp) with primer pair HA-760F and polFragRev. Lane 9, amplification of the N terminal fragment of *NP_PR8_* (1,476 bp, yellow arrow) with primer pair pTIFragFwd and NP-1441R. Lane 10, amplification of the C terminal fragment of *NP_PR8_* (476 bp) with primer pair NP-1116F and polFragRev. Lane 11, amplification of the N terminal fragment of *NA_PR8_* (940 bp) with primer pair pTIFragFwd and NA 905R. Lane 12, amplification of the C terminal fragment of *NA_PR8_* (697 bp) with primer pair NA 743F and polFragRev. Lane 13, amplification of the N terminal fragment of *M_PR8_* (950 bp) with primer pair pTIFragFwd and M-915R. Lane 14, amplification of the C terminal fragment of *M_PR8_* (313 bp) with primer pair M-741F and polFragRev. Lane 15, amplification of the N terminal fragment of *NS_PR8_* (923 bp) with primer pair pTIFragFwd and NS-887R. Lane 16 amplification of the C terminal fragment of *NS_PR8_* (468 bp) with primer pair NS-469F and polFragRev. PCR conditions were similar to those described in SFig 2. B) Full-length PR8 PCR amplicons. Overlapping PCR products generated in A) were mixed at a concentration of 10 ng (each product) and amplified with the forward primer pT1FragFwd and the reverse primer hpol1Rev as described in the main text. The thermal profile was: denaturation at 98°C for 30 sec, 30 cycles of 98°C for 8 sec, 56°C for 2 min, and 72°C for 4 min, and then extension at 72°C for 10 min. All the PCR products were amplified using the Phusion high-fidelity PCR master mix with GC Buffer. The final overlapping PCR amplicons were designated as *pol1PB2_PR8_t1* (2,598 bp, lane 1, yellow arrow), *pol1PA_PR8_t1* (2,490 bp, lane 2, yellow arrow), *pol1NP_PR8_t1* (1,822 bp, lane 3), *pol1NA_PR8_t1* (1,670 bp, lane 4), *po1M_PR8_t1* (1,284 bp, lane 5), and *pol1NS_PR8_t1* (1,147 bp, lane 6), *pol1PB1_PR8_t1* (2,598 bp, lane 7), and *pol1HA_PR8_t1* (2,032 bp, lane 8). M, GeneRuler™ 1 kb plus DNA Ladder.
(JPG)

Figure S4 **HA PCR amplicons flanked with k9pol1 promoter.** Two produce overlapping PCR products for *HA*_Δ*072*_ and *HA*_Δ*VN1203*_ gene segments. PCR conditions used were similar to those described in SFig 2 and in the main text. Lane 1, the k9pol1 promoter (351 bp) was amplified from the pGD2007 vector using the primer pair k9pol1F and k9pol1R. Lane 2, PCR fragment containing the N-terminus of *HA*_Δ*072*_ and the k9t1 signal (36 bp) was produced with the primer pair kTIUni12F and IndoH5-clvR with a size of 1,091 bp. Lane 3, PCR fragment containing the C-terminus of *HA*_Δ*072*_ (760 bp) produced with the primer pair IndoH5-clvF and kPolUTRR. Lane 4, PCR fragment containing the N-terminus of *HA*_Δ*VN1203*_ (1,091 bp) amplified as in lane 2. Lane 5 PCR fragment containing the C-terminus of *HA*_Δ*VN1203*_ (760 bp) produced as in lane 3. Lane 6, the two overlapping *HA*_Δ*072*_ PCR products and the k9pol1 PCR fragment were mixed at a concentration of 10 ng (each product) to generate the full length of *k9pol1HA*_Δ*072*_ *t1* PCR amplicon (2,144 bp) using the primer pair kTIUni12F and k9pol1R. Lane 7, the two overlapping *HA*_Δ*VN1203*_ PCR products and the k9pol1 PCR fragment were mixed at a concentration of 10 ng each and amplified to generate the full length *k9pol1HA*_Δ*VN1203*_ *t1* amplicon (2,144 bp) using the primer pair kTIUni12F and k9pol1R. Lane 8, same as in lane 6, except that *k9pol1HA*_Δ*072*_ *utr* (2, 109 bp) lacks the k9t1 signal after amplification with the primer pair Bm-HA-1F and k9pol1R. Lane 9, same as in lane 7, except tha*t k9pol1HA*_Δ*VN1203*_ *utr* (2,109 bp) lacks the k9t1 signal after amplification. M, GeneRuler™ 1 kb Plus DNA Ladder.
(JPG)

Table S1 **Primer set for production of overlapping Flu PCR amplicons.**
(DOC)

Table S2 **HA sequences from H5Δ_072_ PCR: 7PR8 plaque-purified viruses.**
(DOC)
