# Supplementary material for: Partial and Full PCR-Based Reverse Genetics Strategy for Influenza Viruses
Source: PLoS One. 2012 Sep 28;7(9):e46378. doi: 10.1371/journal.pone.0046378 (PMC3460856; doi:10.1371/journal.pone.0046378)
Supplement: Materials and Methods S1 — Enzymes and kits used to generate full-length Flu PCR amplicons. (DOC) [file pone.0046378.s001.doc]

**Supplementary materials and methods**

**Enzymes and kits used to generate full-length Flu PCR amplicons.**

The enzymes listed below were compared in order to determine how efficiently they could amplify the pol1 promoter sequences as well as the influenza gene of interest under the same PCR conditions:

1. Platinum® Pfx DNA Polymerase (Invitrogen)
2. AccuPrime™ Taq DNA Polymerase High Fidelity (Invitrogen)
3. AccuPrime™ GC-Rich DNA Polymerase (Invitrogen)
4. PfuUltra Hotstart DNA Polymerase (Agilent Technologies Inc, Santa Clara, CA)
5. Cloned Pfu DNA Polymerase AD (Agilent)
6. Roche Expand High Fidelity PCR System dNTP Pack (Roche Applied Science, Indianapolis, IN)
7. Phusion® High-Fidelity PCR Master Mix with GC Buffer (NEB)
8. GoTaq (Promega, Madison, WI)

PCR conditions were adjusted following recommendations from the manufacturers. Based on PCR product yield for fragments and full-length products, we chose the Phusion® High-Fidelity PCR Master Mix with GC Buffer (NEB) as the enzyme and buffer of choice.

**Supplementary Information Legends**

**Figure S1. Pol1- and pol2-driven PCR amplicons.** A) Generation of Flu EGFP replicons from pHW72EGFP. Lane 1, pol1EGFPt1 amplicon amplified with the primer pair pT1FragFwd hpol1Rev. The Flu EGFP amplicon (1103 bp) contained the Flu EGFP replicon (846 bp) flanked by the human pol1 (222 bp) and mouse t1 (35 bp) sequences. Lane 2, pol1EGFPutr amplicon (1068 bp, lacking the t1 sequence) produced with the primer pair Bm-M-1F and hpol1Rev. Lane 3, UTREGFPutr amplicon (846 bp, lacking the pol1 and t1 sequences) amplified with the primers Bm-M-1F and Bm-M-1043R. Lane 4, UTREGFPt1 amplicon (881 bp, lacking pol1 sequence) generated with the primers pT1FragFwd and pol1FragRev. B) Pol2 Flu PCR amplicons produced from pcDNA762 (PB2), pcDNA774 (PB1), pcDNA787 (PA) and pcDNA693 (NP), respectively using the primer pair *pCMVF* and *pBGHR*. Each pol2 Flu PCR amplicon contained the cytomegalovirus immediate early promoter sequence (CMV, 659 bp), the bovine growth hormone polyA signal (BGHpA, 228 bp) and additional non coding regions present within the multiple cloning site of pcDNA3 (Invitrogen). Lane 1, *pol2PB2bgh* (3,386 bp); lane 2, *pol2PB1bgh* (3,385 bp); lane 3,  *pol2PAbgh* (3,271 bp); and lane 4 *pol2NPbgh* (2,603 bp). “M” in panels A and B corresponds to DNA molecular weight marker (GeneRuler™ 1 kb Plus DNA Ladder, Fermentas).

**Figure S2. Generation of HA and NA amplicons.** cDNAs from the H1N1pdm and H5N1 072 viruses were prepared as described in the main text. M, GeneRuler™ 1 kb plus DNA Ladder. Lane 1, unspecific PCR products obtained using one-step RT-PCR to generate the full length of *HA_pdm_* gene (1,840 bp) with the primers pT1HF and polHR. Lane 2, the N terminus of *HA_pdm_* specific PCR product (998 bp) obtained using the primer pair pT1FragFwd, which incorporates the t1 signal, and SwHA-931R. Lane 3, the C terminus of overlapping *HA_pdm_* specific PCR product (1,022 bp) using the primer pair SwHA-752F and polFragRev. Lane 4, the N terminus of *NA_pdm_* specific PCR product (799 bp) obtained using the primer pair pTIFragFwd and SwNA-763R. Lane 5, the C terminus of *NA_pdm_* specific PCR product (924 bp) from N1-562F and polFragRev primer set. Lane 6, the first *HA∆_072_* specific PCR fragment (1,090 bp) obtained with the primers pTIFragFwd and IndoH5-clvR. Lane 7, the second *HA_∆072_* specific PCR fragment (762 bp) obtained with the primers pair IndoH5-clvF and pol1FragRev. Lane 8, the full-length *NA_∆072_* amplicon (1,460 bp) generated with the primer set hTIN1Fwd and polN1Rev. The 25 µl PCR reaction mixture contained 10 ng of cDNAs, 12.5 µl of Master PCR mix, 0.6 µl 100% DMSO, and 10 pmol/µl of each primer. The PCR reaction conditions were 98°C for 30 sec, and then 30 cycles at 98°C for 8 s, 56°C for 1 sec and 72°C for 2 min, ending with 72°C for 10 min. PCR products were amplified using the Phusion high-fidelity PCR master mix with GC Buffer.

**Figure S3. Full-length PCR amplicons from PR8 virus gene segments.** A) The PR8 virus gene segments were amplified as two overlapping PCR fragments, which were performed as follows: Lane 1, amplification of the N terminal fragment of *PB2_PR8_* (1,846 bp) with primer pair pTIFragFwd and PB2-1811R. Lane 2, amplification of the C terminal fragment of *PB2_PR8_* (741 bp, yellow arrow) with primer pair PB2-1643F and polFragRev. Lane 3, amplification of the N terminal fragment of *PB1_PR8_* (1,566 bp) with primer pair pTIFragFwd and PB1-1531R. Lane 4, amplification of the C terminal fragment of *PB1_PR8_* (1,128 bp) with primer pair PB1-1240F and polFragRev. Lane 5, amplification of the N terminal fragment of *PA_PR8_* (1,349 bp) with primer pair pTIFragFwd and PA-1314R. Lane 6, amplification of the C terminal fragment of *PA_PR8_* (1,368 bp) with primer pair PA-892F and polFragRev. Lane 7, amplification of the N terminal fragment of *HA_PR8_* (1,309 bp) with primer pair pTIFragFwd and HA1274R. Lane 8, amplification of the C terminal fragment of *HA_PR8_* (1,042 bp) with primer pair HA-760F and polFragRev. Lane 9, amplification of the N terminal fragment of *NP_PR8_* (1,476 bp, yellow arrow) with primer pair pTIFragFwd and NP-1441R. Lane 10, amplification of the C terminal fragment of *NP_PR8_* (476 bp) with primer pair NP-1116F and polFragRev. Lane 11, amplification of the N terminal fragment of *NA_PR8_* (940 bp) with primer pair pTIFragFwd and NA 905R. Lane 12, amplification of the C terminal fragment of *NA_PR8_* (697 bp) with primer pair NA 743F and polFragRev. Lane 13, amplification of the N terminal fragment of *M_PR8_* (950 bp) with primer pair pTIFragFwd and M-915R. Lane 14, amplification of the C terminal fragment of *M_PR8_* (313 bp) with primer pair M-741F and polFragRev. Lane 15, amplification of the N terminal fragment of *NS_PR8_* (923 bp) with primer pair pTIFragFwd and NS-887R. Lane 16 amplification of the C terminal fragment of *NS_PR8_* (468 bp) with primer pair NS-469F and polFragRev. PCR conditions were similar to those described in SFig 2. B) Full-length PR8 PCR amplicons. Overlapping PCR products generated in A) were mixed at a concentration of 10ng (each product) and amplified with the forward primer pT1FragFwd and the reverse primer hpol1Rev as described in the main text. The thermal profile was: denaturation at 98 ºC for 30 sec, 30 cycles of 98 ºC for 8 sec, 56ºC for 2min, and 72 ºC for 4 min, and then extension at 72 ºC for 10min. All the PCR products were amplified using the Phusion high-fidelity PCR master mix with GC Buffer. The final overlapping PCR amplicons were designated as *pol1PB2_PR8_t1* (2,598 bp, lane 1, yellow arrow), *pol1PA_PR8_t1* (2,490 bp, lane 2, yellow arrow), *pol1NP_PR8_t1* (1,822 bp, lane 3), *pol1NA_PR8_t1* (1,670 bp, lane 4), *po1M_PR8_t1* (1,284 bp, lane 5), and *pol1NS_PR8_t1* (1,147 bp, lane 6), *pol1PB1_PR8_t1* (2,598 bp, lane 7), and *pol1HA_PR8_t1* (2,032 bp, lane 8). M, GeneRuler™ 1 kb plus DNA Ladder.

**Figure S4. HA PCR amplicons flanked with k9pol1 promoter.** Two produce overlapping PCR products for *HA_∆072_* and *HA_∆VN1203_* gene segments. PCR conditions used were similar to those described in SFig 2 and in the main text. Lane 1, the k9pol1 promoter (351 bp) was amplified from the pGD2007 vector using the primer pair k9pol1F and k9pol1R. Lane 2, PCR fragment containing the N-terminus of *HA_∆072_* and the k9t1 signal (36 bp) was produced with the primer pair kTIUni12F and IndoH5-clvR with a size of 1,091 bp. Lane 3, PCR fragment containing the C-terminus of *HA_∆072_* (760 bp) produced with the primer pair IndoH5-clvF and kPolUTRR. Lane 4, PCR fragment containing the N-terminus of *HA_∆VN1203_* (1,091 bp) amplified as in lane 2. Lane 5 PCR fragment containing the C-terminus of *HA_∆VN1203_* (760 bp) produced as in lane 3. Lane 6, the two overlapping *HA_∆072_* PCR products and the k9pol1 PCR fragment were mixed at a concentration of 10ng (each product) to generate the full length of *k9pol1HA_∆072_t1* PCR amplicon (2,144 bp) using the primer pair kTIUni12F and k9pol1R. Lane 7, the two overlapping *HA_∆VN1203_* PCR products and the k9pol1 PCR fragment were mixed at a concentration of 10ng each and amplified to generate the full length *k9pol1HA_∆VN1203_t1* amplicon (2,144 bp) using the primer pair kTIUni12F and k9pol1R. Lane 8, same as in lane 6, except that *k9pol1HA_∆072_utr* (2, 109 bp) lacks the k9t1 signal after amplification with the primer pair Bm-HA-1F and k9pol1R. Lane 9, same as in lane 7, except tha*t k9pol1HA_∆VN1203_utr* (2,109 bp) lacks the k9t1 signal after amplification. M, GeneRuler™ 1 kb Plus DNA Ladder.
