# Supplementary figures and images for: Partial and Full PCR-Based Reverse Genetics Strategy for Influenza Viruses
Source: PLoS One. 2012 Sep 28;7(9):e46378. doi: 10.1371/journal.pone.0046378 (PMC3460856; doi:10.1371/journal.pone.0046378)

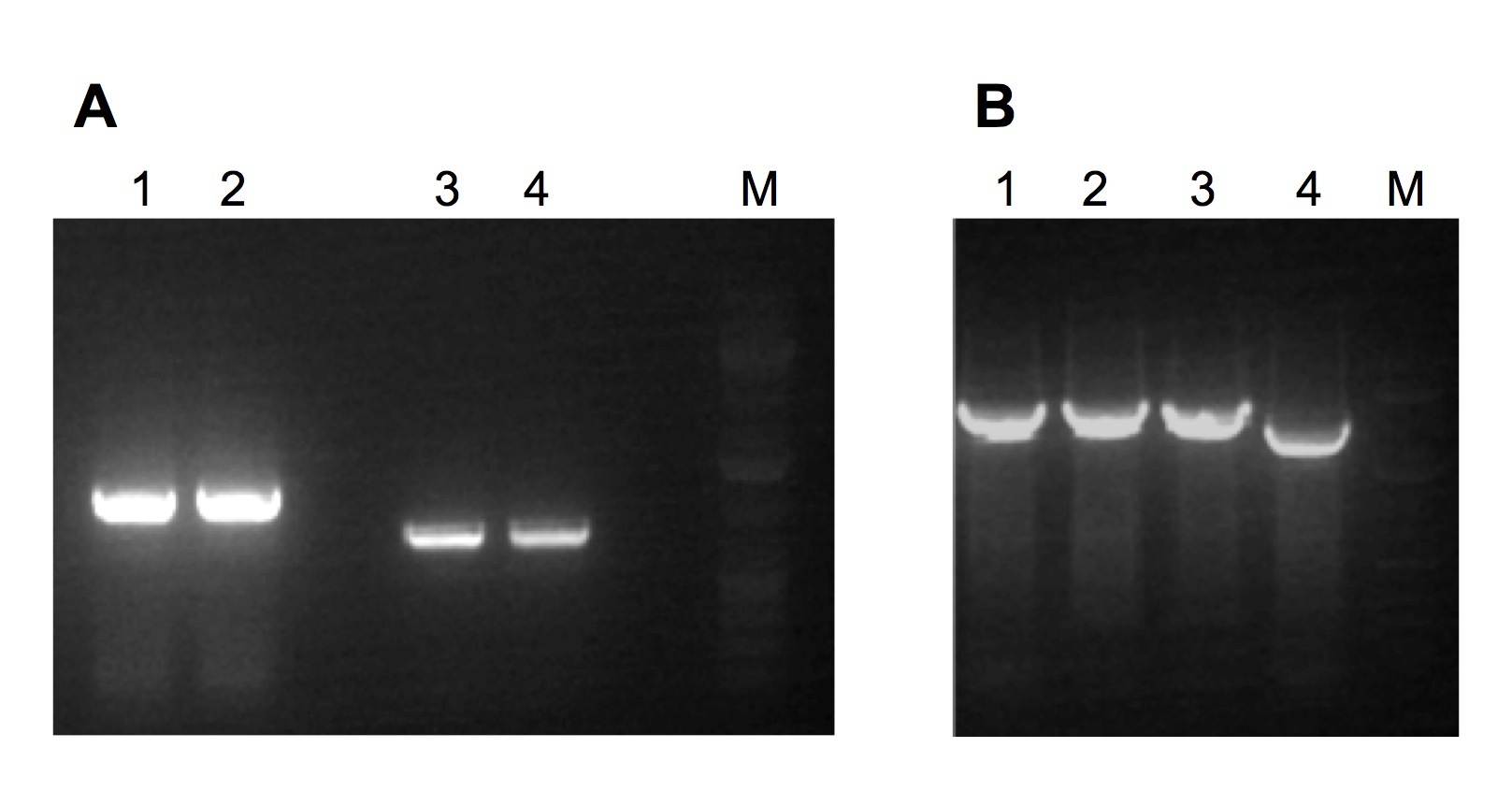

Supplement: Figure S1 — Pol1- and pol2-driven PCR amplicons. A) Generation of Flu EGFP replicons from pHW72EGFP. Lane 1, pol1EGFPt1 amplicon amplified with the primer pair pT1FragFwd hpol1Rev. The Flu EGFP amplicon (1103 bp) contained the Flu EGFP replicon (846 bp) flanked by the human pol1 (222 bp) and mouse t1 (35 bp) sequences. Lane 2, pol1EGFPutr amplicon (1068 bp, lacking the t1 sequence) produced with the primer pair Bm-M-1F and hpol1Rev. Lane 3, UTREGFPutr amplicon (846 bp, lacking the pol1 and t1 sequences) amplified with the primers Bm-M-1F and Bm-M-1043R. Lane 4, UTREGFPt1 amplicon (881 bp, lacking pol1 sequence) generated with the primers pT1FragFwd and pol1FragRev. B) Pol2 Flu PCR amplicons produced from pcDNA762 (PB2), pcDNA774 (PB1), pcDNA787 (PA) and pcDNA693 (NP), respectively using the primer pair pCMVF and pBGHR. Each pol2 Flu PCR amplicon contained the cytomegalovirus immediate early promoter sequence (CMV, 659 bp), the bovine growth hormone polyA signal (BGHpA, 228 bp) and additional non coding regions present within the multiple cloning site of pcDNA3 (Invitrogen). Lane 1, pol2PB2bgh (3,386 bp); lane 2, pol2PB1bgh (3,385 bp); lane 3, pol2PAbgh (3,271 bp); and lane 4 pol2NPbgh (2,603 bp). “M” in panels A and B corresponds to DNA molecular weight marker (GeneRuler™ 1 kb Plus DNA Ladder, Fermentas). (JPG) [file pone.0046378.s002.jpg]

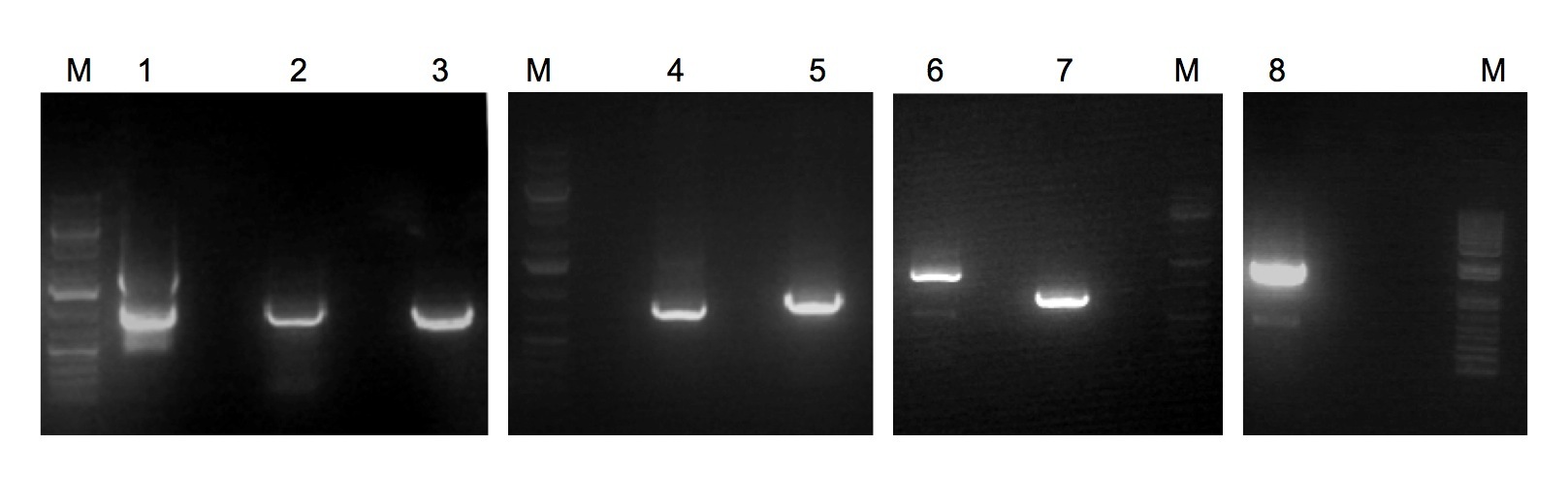

Supplement: Figure S2 — Generation of HA and NA amplicons. cDNAs from the H1N1pdm and H5N1 072 viruses were prepared as described in the main text. M, GeneRuler™ 1 kb plus DNA Ladder. Lane 1, unspecific PCR products obtained using one-step RT-PCR to generate the full length of HApdm gene (1,840 bp) with the primers pT1HF and polHR. Lane 2, the N terminus of HApdm specific PCR product (998 bp) obtained using the primer pair pT1FragFwd, which incorporates the t1 signal, and SwHA-931R. Lane 3, the C terminus of overlapping HApdm specific PCR product (1,022 bp) using the primer pair SwHA-752F and polFragRev. Lane 4, the N terminus of NApdm specific PCR product (799 bp) obtained using the primer pair pTIFragFwd and SwNA-763R. Lane 5, the C terminus of NApdm specific PCR product (924 bp) from N1-562F and polFragRev primer set. Lane 6, the first HAΔ072 specific PCR fragment (1,090 bp) obtained with the primers pTIFragFwd and IndoH5-clvR. Lane 7, the second HA Δ072 specific PCR fragment (762 bp) obtained with the primers pair IndoH5-clvF and pol1FragRev. Lane 8, the full-length NA Δ072 amplicon (1,460 bp) generated with the primer set hTIN1Fwd and polN1Rev. The 25 µl PCR reaction mixture contained 10 ng of cDNAs, 12.5 µl of Master PCR mix, 0.6 µl 100% DMSO, and 10 pmol/µl of each primer. The PCR reaction conditions were 98°C for 30 sec, and then 30 cycles at 98°C for 8 s, 56°C for 1 sec and 72°C for 2 min, ending with 72°C for 10 min. PCR products were amplified using the Phusion high-fidelity PCR master mix with GC Buffer. (JPG) [file pone.0046378.s003.jpg]

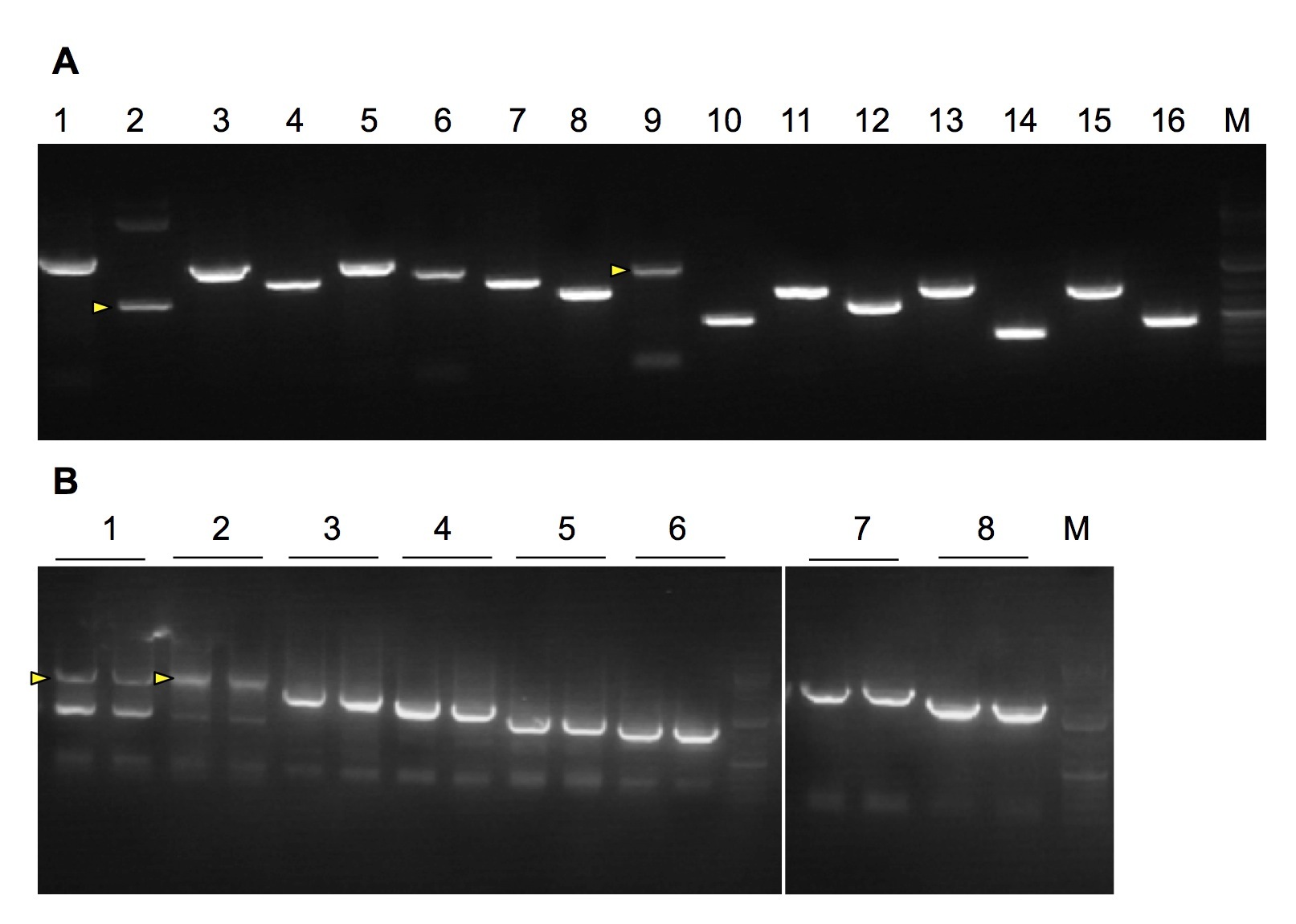

Supplement: Figure S3 — Full-length PCR amplicons from PR8 virus gene segments. A) The PR8 virus gene segments were amplified as two overlapping PCR fragments, which were performed as follows: Lane 1, amplification of the N terminal fragment of PB2PR8 (1,846 bp) with primer pair pTIFragFwd and PB2-1811R. Lane 2, amplification of the C terminal fragment of PB2PR8 (741 bp, yellow arrow) with primer pair PB2-1643F and polFragRev. Lane 3, amplification of the N terminal fragment of PB1PR8 (1,566 bp) with primer pair pTIFragFwd and PB1-1531R. Lane 4, amplification of the C terminal fragment of PB1PR8 (1,128 bp) with primer pair PB1-1240F and polFragRev. Lane 5, amplification of the N terminal fragment of PAPR8 (1,349 bp) with primer pair pTIFragFwd and PA-1314R. Lane 6, amplification of the C terminal fragment of PAPR8 (1,368 bp) with primer pair PA-892F and polFragRev. Lane 7, amplification of the N terminal fragment of HAPR8 (1,309 bp) with primer pair pTIFragFwd and HA1274R. Lane 8, amplification of the C terminal fragment of HAPR8 (1,042 bp) with primer pair HA-760F and polFragRev. Lane 9, amplification of the N terminal fragment of NPPR8 (1,476 bp, yellow arrow) with primer pair pTIFragFwd and NP-1441R. Lane 10, amplification of the C terminal fragment of NPPR8 (476 bp) with primer pair NP-1116F and polFragRev. Lane 11, amplification of the N terminal fragment of NAPR8 (940 bp) with primer pair pTIFragFwd and NA 905R. Lane 12, amplification of the C terminal fragment of NAPR8 (697 bp) with primer pair NA 743F and polFragRev. Lane 13, amplification of the N terminal fragment of MPR8 (950 bp) with primer pair pTIFragFwd and M-915R. Lane 14, amplification of the C terminal fragment of MPR8 (313 bp) with primer pair M-741F and polFragRev. Lane 15, amplification of the N terminal fragment of NSPR8 (923 bp) with primer pair pTIFragFwd and NS-887R. Lane 16 amplification of the C terminal fragment of NSPR8 (468 bp) with primer pair NS-469F and polFragRev. PCR conditions were similar to those descr [file pone.0046378.s004.jpg]

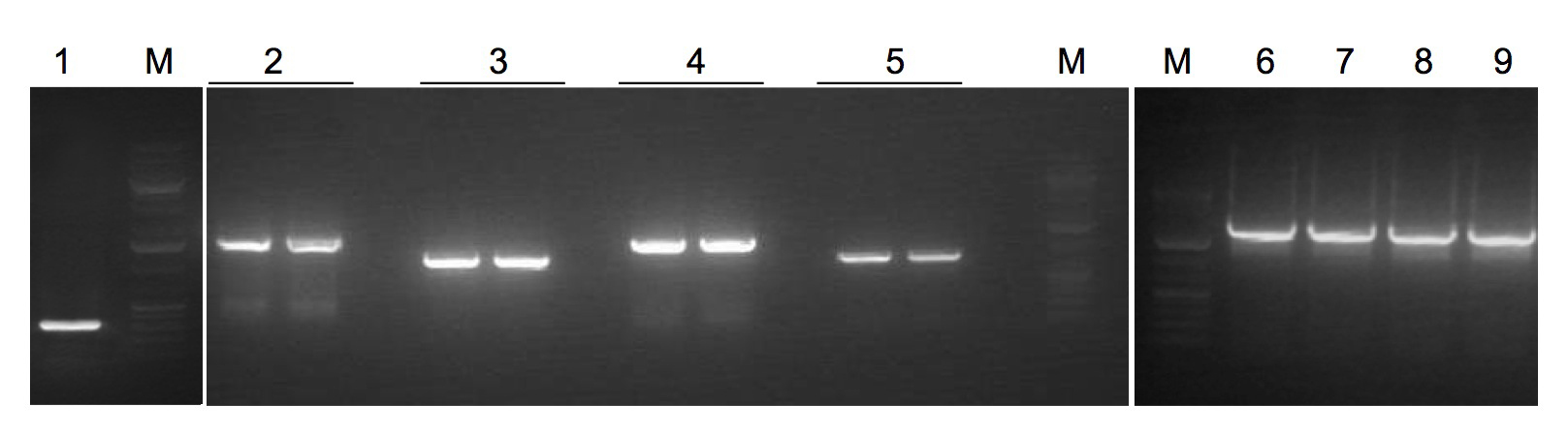

Supplement: Figure S4 — HA PCR amplicons flanked with k9pol1 promoter. Two produce overlapping PCR products for HA Δ072 and HA ΔVN1203 gene segments. PCR conditions used were similar to those described in SFig 2 and in the main text. Lane 1, the k9pol1 promoter (351 bp) was amplified from the pGD2007 vector using the primer pair k9pol1F and k9pol1R. Lane 2, PCR fragment containing the N-terminus of HA Δ072 and the k9t1 signal (36 bp) was produced with the primer pair kTIUni12F and IndoH5-clvR with a size of 1,091 bp. Lane 3, PCR fragment containing the C-terminus of HA Δ072 (760 bp) produced with the primer pair IndoH5-clvF and kPolUTRR. Lane 4, PCR fragment containing the N-terminus of HA ΔVN1203 (1,091 bp) amplified as in lane 2. Lane 5 PCR fragment containing the C-terminus of HA ΔVN1203 (760 bp) produced as in lane 3. Lane 6, the two overlapping HA Δ072 PCR products and the k9pol1 PCR fragment were mixed at a concentration of 10 ng (each product) to generate the full length of k9pol1HA Δ072 t1 PCR amplicon (2,144 bp) using the primer pair kTIUni12F and k9pol1R. Lane 7, the two overlapping HA ΔVN1203 PCR products and the k9pol1 PCR fragment were mixed at a concentration of 10 ng each and amplified to generate the full length k9pol1HA ΔVN1203 t1 amplicon (2,144 bp) using the primer pair kTIUni12F and k9pol1R. Lane 8, same as in lane 6, except that k9pol1HA Δ072 utr (2, 109 bp) lacks the k9t1 signal after amplification with the primer pair Bm-HA-1F and k9pol1R. Lane 9, same as in lane 7, except that k9pol1HA ΔVN1203 utr (2,109 bp) lacks the k9t1 signal after amplification. M, GeneRuler™ 1 kb Plus DNA Ladder. (JPG) [file pone.0046378.s005.jpg]
