# Supplementary material for: Partial and Full PCR-Based Reverse Genetics Strategy for Influenza Viruses
Source: PLoS One. 2012 Sep 28;7(9):e46378. doi: 10.1371/journal.pone.0046378 (PMC3460856; doi:10.1371/journal.pone.0046378)
Supplement: Table S1 — Primer set for production of overlapping Flu PCR amplicons. (DOC) [file pone.0046378.s006.doc]

**Table 1S. Primer set for production of overlapping Flu PCR amplicons**

| **Name*** | **Sequences (5’-3’)** |
| --- | --- |
| Uni12 | AGCAAAAGCAGG |
| UTR-H1Fwd | agcaaaagcaggggataattgaatc |
| UTR-H1Rev | tagtagaaacaagggtgttttttccgt |
| hT1FragFwd | accggagtactggtcgacctccgaagttggggggg*AGCAAAAGCAGG* |
| pT1HF | accggagtactggtcgacctccgaagttggggggg*agcaaaagcagggg* |
| SwHA-931R | TCTGAAATGGGAGGCTGGTGTT |
| SwHA-752F | TAGAGCCGGGAGACAAAATAACAT |
| polHR | gtcggcattttgggccgccgggttatt*agtagaaacaagggtgtttt* |
| pol1FragRev | gtcggcattttgggccgccgggttatt*agtagaaacaagg* |
| UTR-N1fwd | agcaaaagcaggagtttaaaatg |
| UTR-N1Rev | tagtagaaacaaggagtttttttca |
| hT1N1Fwd | accggagtactggtcgacctccgaagttggggggg*AGCAAAAGCAGGAGT* |
| N1-562 F | CAAGTGCTTGTCATGATGGCA |
| SwNA-763R | TTACTTGGTCCATCGGTCATTACA |
| polN1Rev | gtcggcattttgggccgccgggttattagtagaaacaaggagtttttttca |
| IndoH5-clvF | ACAGCCCTCAA ACTGAAACTAGA GGACTATTTGGAGCTATAG |
| IndoH5-clvR | TCTAGTTTCAGT TTGAGGGCTGTTTCTGAGCC |
| polF | aataacccggcggcccaaaatgccgac |
| hPolIRev | atgctgacaacgtccccggcccggcgctgct |
| k9pol1F | ACCTACCTGGCAACAAAAAATGTT |
| k9pol1R | GCCTGCCTCCGGAGAACTTTG |
| kTIUni12F | GCGGGGACAGCTGGTCGACCGGATCCACCAGGAGGG*AGCAAAAGCAGG* |
| kPolUTRR | AACATTTTTTGTTGCCAGGTAGGTagtagaaacaagg |
| PB2-1643F | TCAATGATGTGGGAGATTAA |
| PB2-1811R | AACCCACTGTATTGGCCTCTAATGGC |
| PB1-1240F | GGAATGATGATGGGCATGTT |
| PB1-1531R | GGAAGCTCCATGCTGAAATTGGCA |
| PA-892 F | TTAAGCATTGAGGACCCAAGTCA |
| PA-1314R | GGGTCAGTGAGAGAAAACTCCATGCT |
| HA-760F | TGAACTATTACTGGACCTTGC |
| HA-1274R | GTTGAATTCTTTACCCACAGC |
| NP-1116F | GCTTTCCACTAGAGGAGTTC |
| NP-1441R | GCTCGAAGACTCCCCGCCCCTG |
| NA-743F | CAAGATCGAAAAGGGGAAGGTTAC |
| NA-905R | GAACCATGCCAGTTGTCTCTGC |
| M-741F | CCTATCAGAAACGAATGGGGG |
| M-915R | CTCCTTCCGTAGAAGGCCCTC |
| NS-469F | GGGCTTTCACCGAAGAGGGAG |
| NS-887R | AGAAACAAGGGTGTTTTTTA |
| pCMVF | AGGGCGACACGGAAATGTTGAA |
| pBGHR | cagacaatgcgatgcaatttcctc |
| pTI2F | CTAGCAGTTAACCGGAGTACTGGT |
| hT1FragRev | *CCTGCTTTTGCT*cccccccaacttcggaggtcgaccagtactccggt |
| pDP2066F | ataattctcttactgtcatgccatc |
| pDP2416R | aacatttccgtgtcgcccttattcc |

* Description on the use of the primer set is found in Materials and Methods and in the Supplementary information.
