# Supplementary material for: Partial and Full PCR-Based Reverse Genetics Strategy for Influenza Viruses
Source: PLoS One. 2012 Sep 28;7(9):e46378. doi: 10.1371/journal.pone.0046378 (PMC3460856; doi:10.1371/journal.pone.0046378)
Supplement: Table S2 — HA sequences from H5Δ072 PCR: 7PR8 plaque-purified viruses. (DOC) [file pone.0046378.s007.doc]

**Table 2S. HA sequences from H5∆_072_ PCR: 7PR8 plaque-purified viruses**

| Clones | Mutations in HA sequences^§^ | | HI titer against chicken antisera | | |
| --- | --- | --- | --- | --- | --- |
|  | Amino acids | Nucleotide sequences | Serum 05^†^ | Serum 23^†^ | Serum 40^†^ |
| No.07 | N10S | ^76^AAC^78^→^76^AGC^78^ | 1280 | 2560 | 2560 |
| No.30 | No change | ^411^T→^411^C | 1280 | 2560 | 2560 |
| No.39 | D171G | ^559^GAT^561^→^559^GGT^561^ | 1280 | 1280 | 1280 |
| No.43 | I151T | ^499^ATC^501^→^499^ACC^501^ | 1280 | 1280 | 1280 |
| Others | No change | No change | 2560 | 2560 | 2560 |
| pH5∆_072_* | No change | No change | 2560 | 2560 | 2560 |

^§^ HA sequences from plaque-purified viruses contained a monobasic cleavage site (TETR)

^†^ Chicken sera obtained from chickens immunized with live attenuated ∆H5_072_ vaccine virus in WF10att background.

* Unpurified pH5∆_072_:7PR8 virus obtained with pDP-H5∆_072_ plasmid. No change based on sequence generated from the wild type virus.
